# Evolutionary history exposes radical diversification among classes of interaction partners of the MLLE domain of plant poly(A)-binding proteins

**DOI:** 10.1186/s12862-015-0475-1

**Published:** 2015-09-16

**Authors:** Domingo Jiménez-López, Jaime Bravo, Plinio Guzmán

**Affiliations:** Departamento de Ingeniería Genética, Centro de Investigación y de Estudios Avanzados, Unidad Irapuato, Apartado Postal 629, Irapuato, Gto. 36821 Mexico; Present address: Department of Microbiology and Immunology, University of Texas Medical Branch, Galveston, TX 77555 USA

## Abstract

**Background:**

Poly(A)-binding proteins (PABPs) are evolutionarily conserved proteins that have important functions in the regulation of translation and the control of mRNA stability in eukaryotes. Most PABPs encode a C-terminal domain known as the MLLE domain (previously PABC or CTC), which can mediate protein interactions. In earlier work we identified and predicted that four classes of MLLE-interacting proteins were present in *Arabidopsis thaliana*, which we named CID A, B, C, and D. These proteins encode transcription-activating domains (CID A), the Lsm and LsmAD domains of ataxin-2 (CID B), the CUE and small MutS-related domains (CID C), and two RNA recognition domains (CID D). We recently found that a novel class that lacks the LsmAD domain is present in CID B proteins.

**Results:**

We extended our analysis to other classes of CIDs present in the viridiplantae. We found that novel variants also evolved in classes CID A and CID C. A specific transcription factor domain is present in a distinct lineage in class A, and a variant that lacks at least two distinct domains was also identified in a divergent lineage in class C. We did not detect any variants in Class D CIDs. This class often consists of four to six highly conserved RNA-binding proteins, which suggests that major redundancy is present in this class.

**Conclusions:**

CIDs are likely to operate as components of posttranscriptional regulatory assemblies. The evident diversification of CIDs may be neutral or may be important for plant adaptation to the environment and for acquisition of specific traits during evolution. The fact that CIDs subclasses are maintained in early lineages suggest that a presumed interference between duplicates was resolved, and a defined function for each subclass was achieved.

**Electronic supplementary material:**

The online version of this article (doi:10.1186/s12862-015-0475-1) contains supplementary material, which is available to authorized users.

## Background

Poly(A) binding protein (PABP) binds to the 3′ poly(A) tail of messenger RNA (mRNA) [1]. This protein is evolutionarily conserved across eukaryotes and has expanded in plants as a multigene family [2, 3]. PABPs are involved in most aspects of mRNA biology (translation, stability, export, deadenylation, and biogenesis). PABP domain structure consists of an amino-terminal domain that includes four distinct RNA recognition motifs (RRM1-4), followed by a linker region and a carboxy-terminal domain known as MLLE (formerly CTC or PABC). The MLLE domain binds proteins that contain a poly(A)-binding protein interacting motif 2 (PAM2) [4–6].

PABP is viewed as a translation initiation factor that stimulates translation promoting mRNA circularization [1]. It bridges between the poly(A) and the 5′ cap structure, mediated by the interaction with the eukaryotic translation initiation factor 4G and 4E (eIF4G and eIF4E) complex [7–9]. PABP function can also be modulated within assemblies by the interaction with other factors. Two human proteins, PABP-interacting protein 1 (Paip1) and PABP-interacting protein 2 (Paip2), enhance or suppress translation, respectively, by binding to PABP. Both Paip proteins bind to PABP using two separate motifs; one motif protein corresponds to an acidic segment that binds to the RRM and the other to PAM2, which binds to MLLE [10]. The MLLE domains of the PABPs also mediate the interactions with several other proteins. Some of these proteins are involved with RNA metabolism, such as ataxin-2, eIF4b, eRF3, GW182, HEZL, and LARP4. Other proteins, which may have a role in translation regulation, are MKRN1 and TOB1/2, which encode an E3 ubiquitin-ligase and an anti-proliferative protein that suppresses cell growth, respectively [11, 12]. The MLLE and PAM2 domains are conserved in the plant kingdom, so PABP-interacting proteins have also been described in plants. Proteins that interact with MLLEs have been identified and designated as CIDs (CTC-interacting domains) [13]. Thirteen *Arabidopsis thaliana* CIDs containing distinct domains have been grouped into four classes (A–D). This diversity and abundance of PABP interactors suggests that CIDs may affect translational control in plants [13].

**Fig. 1:**
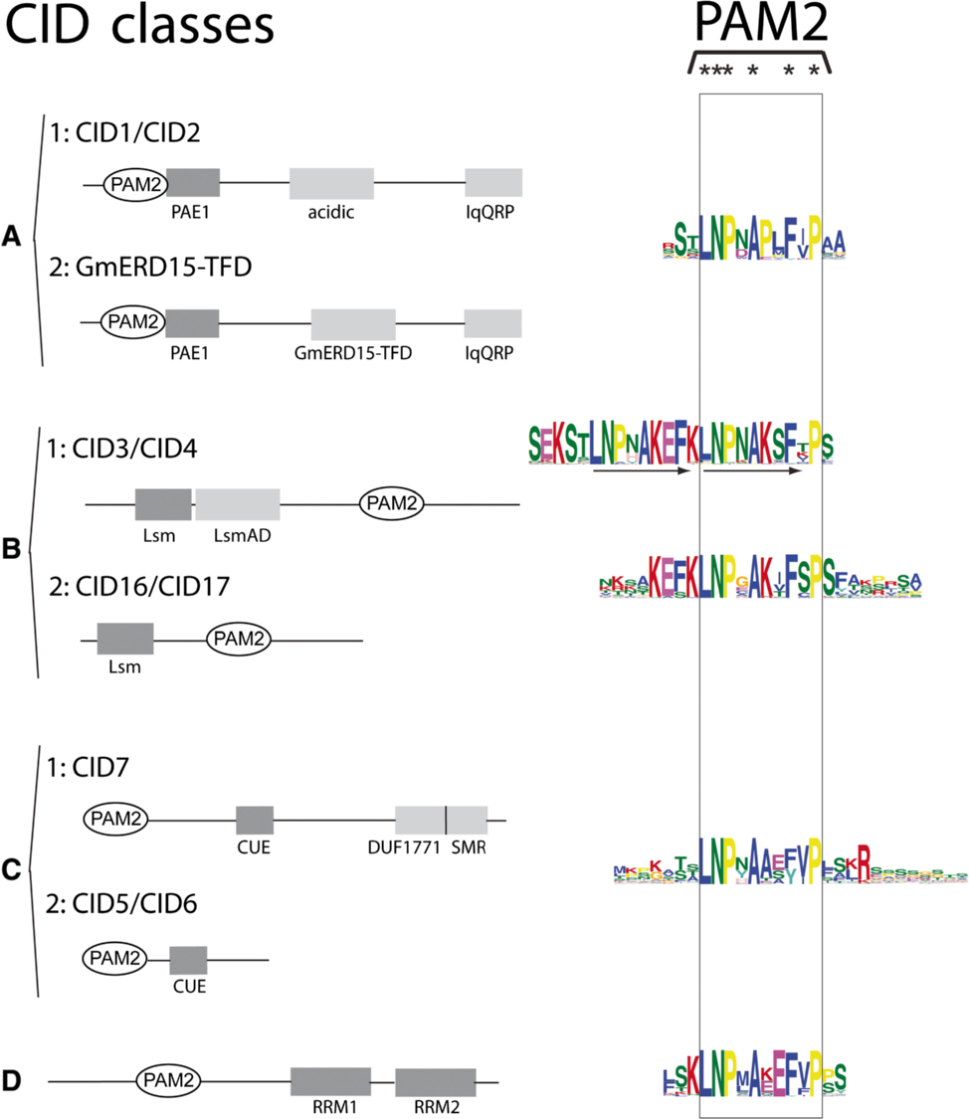
Domain structure of the four classes of CID proteins. Classes are denoted by letters (**a**, **b**, **c** and **d**), and subclasses by letters and a subsequent number (1 or 2). Representing *A. thaliana* CID orthologs defining each subclass or the *G. max* ortholog in subclass A2 are indicated. The schematic representation of relevant domains proposed for each class is depicted. The sequence LOGO for the PAM2 domain generated on each class are aligned together and enclosed by a rectangle; highly conserved residues are denoted at the top by an asterisk. PAE1, PAM2 Associated Element 1 [21]; acidic, rich in acidic amino acids [13]; IqQRP, a highly conserved signature [21]; GmERD15-TFD, Transcription Factor Domain predicted in *G. max* GmERD15 (*Glyma02g42860*) [23]; Lsm, Like RNA splicing domain Sm1 and Sm2, LsmAD, Lsm Associated Domain [18]; CUE, Coupling of Ubiquitin conjugation to ER degradation domain [24]; DUF1771, Domain of Unknown Function; SMR, Small MutS-Related domain [13]; RRM, RNA Recognition Motif [13]

Little is known about the modulation of PABP assemblies in plants. We previously performed a genome-wide survey of group B CIDs (ataxin-2 orthologs) across eukaryotic organisms to further understand the evolution and divergence of CIDs. Class B includes four *A. thaliana* genes (*CID3*, *CID4*, *CID16,* and *CID17*), which are orthologs of the mammalian ataxin-2 and yeast Pbp1 genes [13]. They encode evolutionarily conserved proteins across eukaryotes. These proteins have been implicated in spinocerebellar ataxia type 2 (SCA2) and amyotrophic lateral sclerosis (ALS) in humans, two progressive neurodegenerative diseases. They contain a Like RNA splicing domain Sm1 and Sm2 (Lsm), which potentially binds RNA, and a Like-Sm-associated domain (LsmAD) that includes a clathrin-mediated trans-Golgi signal (Fig. 1). LsmAD also mediates the interaction with the DEAD/H-Box RNA helicase DDX6. CID16 and CID17 are derivatives that lack LsmAD, which suggests that new subcellular distributions and functional properties have developed on these variants. Notably, in both CID3 and CID4 PAM2 is composed of two tandem reiterations that may provide them with alternative functional features (Fig. 1).

The objective of the current study was to continue the analysis by surveying the other three groups of CIDs across plant species. We found that similar to group B, newly evolved functions might have diverged in two additional groups after loosing defined domains. This diversification may be neutral or might represent novel responses to a variety of environmental changes. The presence of these novel gene functions suggests that some CIDs might participate to increase complexity within the PABPs assemblies providing new and refined ways to modulate translation in plants.

## Methods

### CID identification and retrieval

The sequences used in this work were retrieved from 39 Viridiplantae genomes deposited in the Phytozome 10.2 database (http://phytozome.jgi.doe.gov/pz/portal.html#) and from the previous version, Phytozome 9.1. The genomes included 2 basal plant species (a Bryophyte and a Lycophyte), 1 basal angiosperm, 7 monocots, and 28 eudicot plants (species and genes are listed in Additional file 1). Seven truncated sequences were readily assembled into CID-like genes using visual inspection (i.e., *Arabidopsis lyrata 883045*, *Thellungiella halophila Thhalv10012369m*, *Mimulus guttatus v1.1 mgv1a014050m*, *Setaria italica Si014490m*, *Panicum virgatum v0.0 Pavirv00050533m*, *Selaginella moellendorffii 56081*, and *Selaginella moellendorffii 66365*. We performed BLAST searches using the complete polypeptide sequences of the *Arabidopsis thaliana* orthologs *CID1*, *CID5*, *CID7*, and *CID8*.

### Sequence alignments and phylogenetic analysis

Peptide sequence alignments were performed using ClustalX software version 2.0.12 20 [14]. Maximum-likelihood (ML), neighbor-joining (NJ), and maximum-parsimony (MP) phylogenetic trees were generated using MEGA 6 software [15]; 1000 bootstrap replicates were obtained in the NJ and MP analysis. The Jones-Taylor-Thornton model was used with 20 gamma categories and the posterior probabilities support values for each node were computed by resampling 1000 times during the ML estimation. Statistical significance in percentages >50 % for the NJ and maximum-parsimony (MP), and posterior probabilities <0.5 for the maximum-likelihood (ML) are indicated on the nodes. Phylogenetic trees were based on complete protein sequences or on distinct domains that were generally included in a sequence LOGO. For example, the 29 amino acid segment comprising PAE1 (LOGO #A1), the 41 amino acid segment comprising the CUE domain (LOGO #C1), and the 174 amino acid segment comprising both RRM domains were used to generate the trees in classes A, C, and D, respectively. The trees were displayed and edited by iTOL (Interactive Tree Of Life, http://itol.embl.de/) software [16].

### Generation of sequence LOGOs

As previously detected in class B, the apparent occurrence of gene variants within classes of CIDs is a readily noticeable characteristic. We used MEME software (University of Queensland, St. Lucia, Australia) to search for CID classes A, C, and D and discover motifs that might support CID classification and have functional significance. Conserved motifs for each protein class were obtained using Multiple EM for Motif Elicitation (MEME) version 4.9.1 (http://meme.nbcr.net/meme/cgi-bin/meme) [17]. The parameter values of 0 or 1 per sequence, and 6 and 75 amino acids as minimum and maximum sizes of motifs, respectively, were used. The E-value cutoff was < e-10. The sequences that had the characteristics for CID class definition or that contained known domains (PAM2, CUE, SMR, RRM, DUF1771) are presented in Additional file 2.

## Results

### Identification of CIDs across embryophyte species

**Fig. 2:**
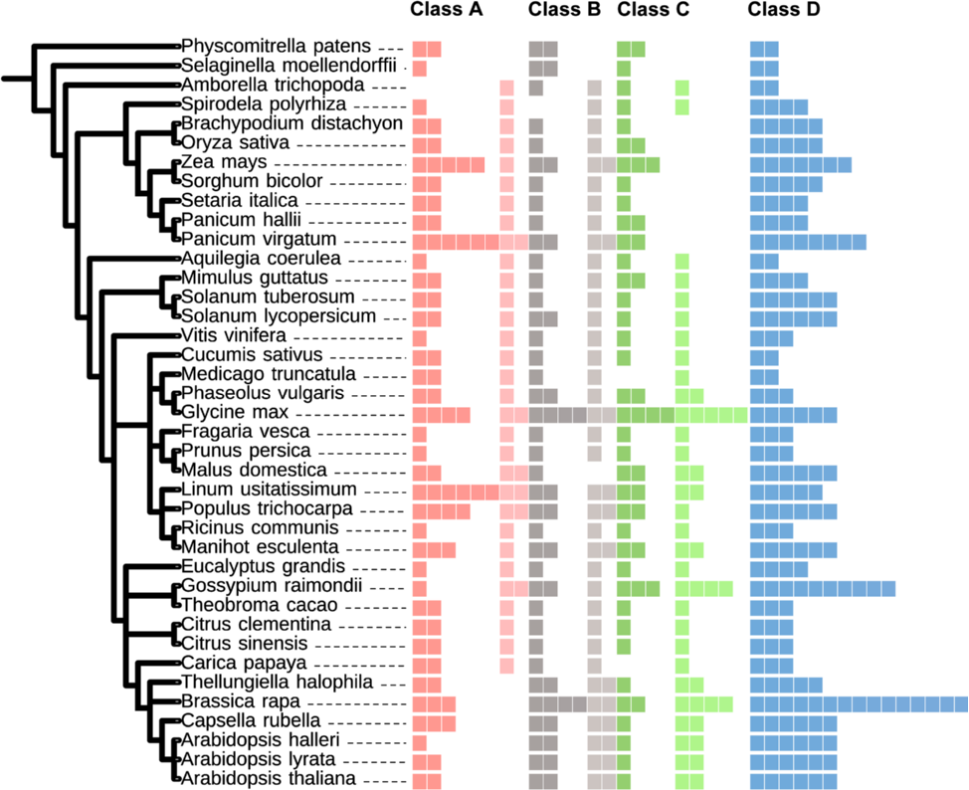
Number of retrieved viridiplantae CID proteins. The phylogenetic relationship between 39 viridiplantae genomes is displayed at the right. Relationships were adapted from the National Center of Biotechnology Information (NCBI) taxonomy server (http://www.ncbi.nlm.nih.gov/Taxonomy) [37]. CID paralogs are indicated by colored squares. Dark and light colors represent CID subclasses 1 and 2, respectively (Fig. 1 and Additional file 1). Class B sequences are from a previous work, except for the orthologs from *Amborella trichopoda*, *Spirodela polyrhiza*, *Panicum halli*, *Malus domestica*, and *Arabidopsis halleri*, which are also listed in Additional file 1

The domain architectures for the four classes of CID proteins are presented in Fig. 1, next to the sequence logos for the PAM2 motif from each class or subclass. Each class has a distinct domain architecture, which includes a conserved PAM2. We previously surveyed for ataxin-2 genes (class B CIDs) across 127 eukaryotic species, including plants, animals, and fungi. Two class B CID subclasses were retrieved. One subclass corresponded to a novel class that does not encode the LsmAD domain (CID16/CID17, Fig. 2). Ataxin-2 genes (Class B) are the only CIDs that are evolutionarily conserved across eukaryotes [18]. CID orthologs of classes A, C, and D were previously only identified in land plants (embryophytes). Hence, we only sampled the embryophyte genes of these three classes of CIDs. Subclasses were considered to be present if a particular domain was determined to set apart a set of peptides from several distinct lineages.

Genes from all classes were retrieved from all the species analyzed, which indicated that their functions have been preserved across land plants. One or two genes were identified in the basal species, the lycophyte *Selaginella moellendorffii* and the moss *Physcomitrella patens*, and in the earliest diverging angiosperm, *Amborella trichopoda*. Conversely, the numbers of genes have increased in some species that have experienced additional or recent genome duplications (*Brassica rapa*, *Glycine max*, *Linum usitatissimum*, *Populus trichocarpa*, *Panicum virgatum*) (Fig. 2; Additional file 1).

### Diversity of transcription factor domains among class A CIDs

Class A genes consist of *A. thaliana* CID1 and CID2. CID1 corresponds to the *EARLY RESPONSIVE TO DEHYDRATION 15* (*ERD15*) gene, which was formerly isolated as cDNA rapidly induced in response to dehydration [19]. In *A. thaliana*, CID1/ERD15 is a component of the stress response, particularly as a negative regulator in the phytohormone abscisic acid (ABA) signaling response. Ectopic expression of CID1/ERD15 reduces ABA sensitivity, and reduction in CID1/ERD15 levels results in ABA hypersensitivity [20, 21]. Functional diversification has been assumed for a soybean *CID1*/*ERD15* homolog, *GmERD15*. This homolog links an osmotic stress-induced cell death signal to endoplasmic reticulum stress by functioning as a transcription factor that binds to the N-rich protein (NRP)-B promoter. Conversely, the *A. thaliana CID1*/*ERD15* homolog does not function as a transcriptional activator at the NRP-B promoter [22, 23]. Analysis of CID1/ERD15 proteins derived mostly from EST clones revealed conserved domain architecture. CIDs from this class are small proteins ranging from 120 to 170 amino acid residues in length containing a conserved motif, PAM2 Associated Element 1 (PAE1), located adjacent to PAM2, an acidic region, and the highly conserved signature IqQRP at the carboxy-terminus (Fig. 1) [21].

In class A, two subclasses were considered to locate apart putative orthologs of the *Glycine max GmERD15* (*Glyma02g42860*): subclasses CID1/CID2 and GmERD15-TDF (see Fig. 1). *Glyma02g42860* was previously described in *G. max* as a transcription factor to encode a specific transcription activation domain not present in *A. thaliana* [23]. We assigned the designation GmERD15-TFD to this domain (Fig. 1). This sequence was identified as a search sequence for LOGOs. Indeed, neither the Brassicaceae nor the two basal species encode this class of ortholog. Accordingly, subclass 1 contains *A. thaliana* CID1/ERD15, CID2 and members that were not identified as putative orthologs of GmERD15.

**Fig. 3:**
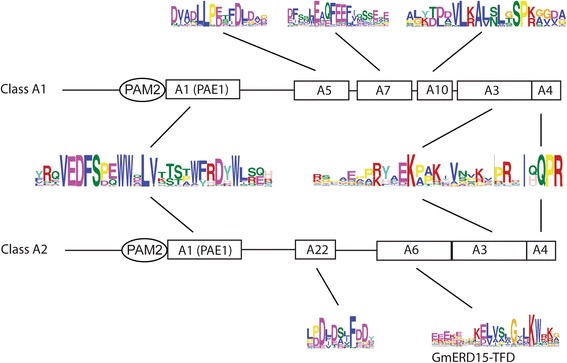
Comparison of the domain architectures of class CID A1 and class CID A2 proteins. Domain architecture is represented, including PAM2, LOGO #A1 (PAE1), LOGO #A3, and LOGO #A4 that were common to class A CIDs. The primary sequence for each LOGO is shown. LOGO #A5, LOGO #A7, and LOGO #A10 were specific for class A1. LOGO #A22 and LOGO #A6 (GmERD15-TFD) were specific for class A2. The alignments of the CID A1 and CID A2 proteins are presented in Additional file 3 and Additional file 4, respectively

MEME searches were performed in a set of 121 CID A protein sequences that varied between 99 and 186 amino acids residues in length. Four sequence LOGOs were common to all or almost all of the polypeptides. LOGO #A2 corresponded to PAM2. LOGO #A1 defined the previously reported PAE1 sequence adjacent to PAM2. These two LOGOs were present in all of the predicted polypeptides. LOGOs #A3 and LOGO #A4 mapped to the carboxy-terminal region and were found in 70 and 80 % in the predicted polypeptides, respectively. LOGO #A4 included the previously described signature IqQRP (Fig. 3). Two subclasses, CID A1 and CID A2, were considered based on the sequence LOGOs mapped between PAE1 and LOGO #A3. This region was previously described as acidic. LOGO #A5 was present in all CID A1 proteins, except for the two basal species *P. patens* and *S. moellendorffii*, and in the Brassicaceae CID2 orthologs (Fig. 3 and Additional file 3, lanes 1–8). Specific sequence LOGOs for eudicot and monocot species also mapped to this region (LOGO #A7 and LOGO #A10, respectively; Fig. 3 and Additional file 3). For the other class, LOGO #A6 and LOGO #A22 mapped to almost all proteins (Fig. 3 and Additional file 3). LOGO #A6 contained the transcriptional activation domain previously described as present in the soybean ortholog, GmERD15 (Glyma02g42860) (Fig. 3). LOGO #A6 was not found in either of the two basal species, *P. patens* or *S. moellendorffii*, nor in the six Brassicaceae species tested (Fig. 3 and Additional file 4). It was present in eudicot and monocot species, including the early lineage *A. trichopoda* and *S. polyrhiza*.

**Fig. 4:**
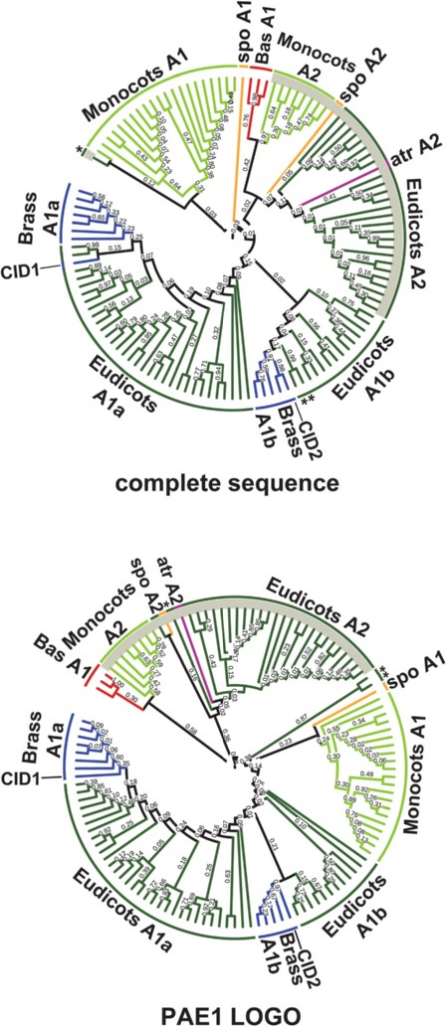
Phylogeny of CID A proteins. The PAE1 sequence LOGO (LOGO #A1) and the complete polypeptide sequence were used to obtain the trees. The topology was generated using the neighbor-joining (NJ) method. CID A2 class is point out in a gray circular line. Branch for groups of organisms were named with the subclass code followed by a letter. Branch color codes: eudicots, dark green; monocots, light green; Brassicaceae (Brass), blue; the basals *Selaginella moellendorffii* and *Physcomitrella patens* (Bas), red; *Amborella trichopoda* (atr), purple; *Spirodela polyrhiza* (spo), mustard yellow. The positions of the two *A. thaliana* paralogs, CID1, and CID2 are shown. (*) are in dissimilar places in both trees: aco|Aquilegia coerulea Aquca 005 00211.1, ccl|Citrus clementina Ciclev10032949m, csi|Citrus sinensis orange1.1g031370m. Species and gene names are as presented in the rectangular phylogeny in Additional file 5. Trees generated using MP and ML are displayed in Additional file 6

Construction of the phylogenetic distribution of class A CIDs was performed using standard approaches. We built phylogenies containing 121 CID A sequences using the NJ, MP, and ML methods. Trees based on complete polypeptide sequences or on the 29 amino acids segment comprising PAE1 (LOGO #A1) were generated; the NJ trees are shown in Fig. 4 and in Additional file 5, and the MP and ML trees in Additional file 6). The phylogenetic trees clustered the LOGO #A6 (GmERD15-TFD)-containing sequences in both types of trees. Minor inconsistencies of sequence location among the two CID A subclasses were detected when comparing the three methods. The sequence Bdi|*Brachypodium distachyon* Bradi1g18780.1 was misplaced in both trees generated by the ML method and in the tree based on the PAE1 LOGO by the MP method. In addition, one of the *Spirodela polyrhiza* sequences was misplaced in the tree based on complete sequences and generated by the MP methods (see spoA2 in Additional file 6). The GmERD15-TFD-containing clade contained members for every species analyzed (encircled in gray in Fig. 4), except the six Brassicaceae, and the two basal, species (blue and red clades in Fig. 4). Accordingly, the orthologs of this *G. max* paralog (GmERD15-TFD-containing sequences) were absent in the six species of Brassicaceae tested (Fig. 2). *A. thaliana* CID1 and CID2 grouped in separate clades, suggesting that they belong in two distinct lineages (blue branches in Fig. 4).

### Loss of domains after duplication on maintained class C CIDs in eudicots

Class C consists of three genes (*CID5*, *CID6*, *CID7*), which encode a variant of the Coupling of Ubiquitin conjugation to ER degradation (CUE) domain that binds ubiquitin in *A. thaliana* [13, 24]. CID5 and CID6 are highly related proteins. CID7 also carries a Small MutS-Related (SMR) domain and the domain of unknown function DUF1771 (Fig. 1). MutSs are found in enzymes involved in mismatch repair of DNA bases, and the SMR domain is often present in bacterial and eukaryotic proteomes [25]. CID5 corresponds to the *INCREASED POLYPLOIDY LEVEL IN DARKNESS 1-1D* (*IPD1-1D*) gene. The ipd1-1D mutant was identified as an activation tagging line that displays increased polyploidy in dark-grown hypocotyl cells. CID5/IPD1 may be a component of the machinery that controls endoreplication cell cycles [24].

Orthologs from subclass C1 were retrieved from all species, except *Carica papaya* and *Medicado truncatula*. They were found mostly as one or two copies, but four were found in *G. max*. Conversely, subclass C2 that includes orthologs of CID5/CID6 were only present in eudicots and ranged from one to five copies (Fig. 2). The two copies retrieved from *Spirodela polyrhiza* are likely to be partial or rearranged sequences; they both clustered within subclass C1 in a phylogenetic tree (see below).

**Fig. 5:**
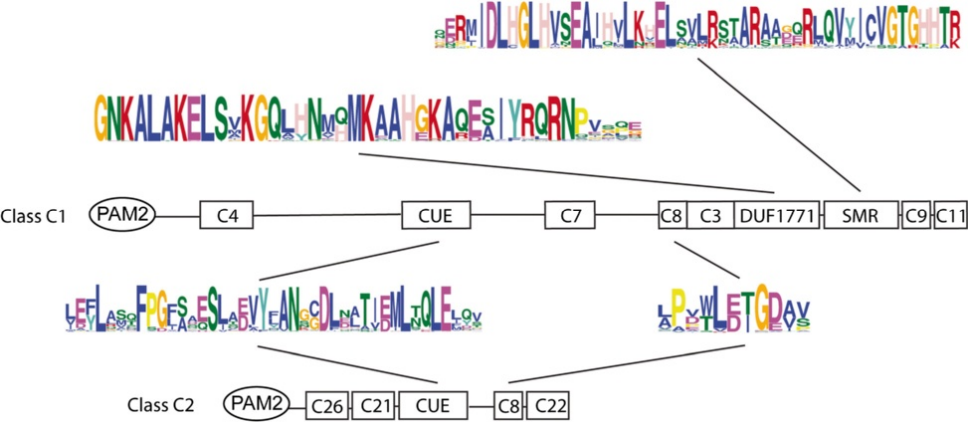
Comparison of the domain architectures of class CID C1 and class CID C2 proteins. The locations of previously identified domains are indicated: PAM2, CUE, DUF1771, SMR. Relevant sequence LOGOs are represented by a number. LOGOs containing DUF1771, SMR, and CUE and LOGO #C8 are displayed; the catalog of sequence LOGOs is presented in Additional file 2

**Fig. 6:**
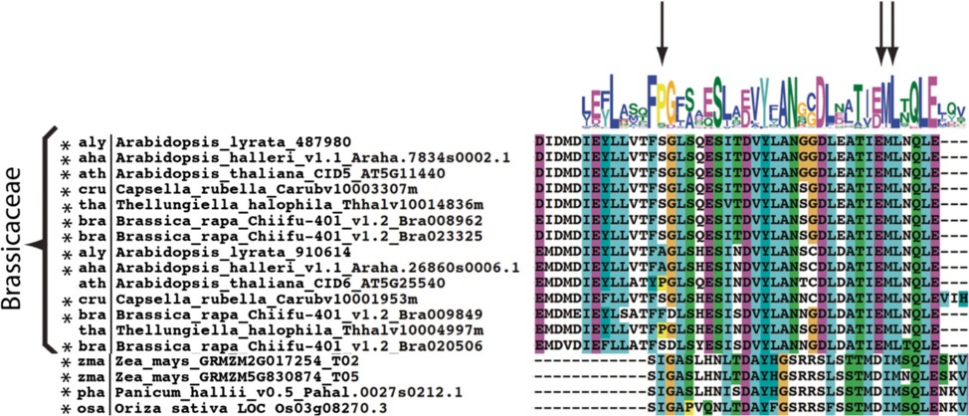
Alignment of the CUE motif of CID C proteins. The alignment region encompassing sequence LOGO #1 of Brassicaceae CID C2 sequences and of four CID C1 monocots is displayed; the alignment of all CID C sequences is shown in Additional file 8. The locations of important residues are highlighted by arrows (the invariant proline residue and a di-leucine motif). (*) indicates substitution of the invariant proline residue

MEME searches were performed for a set of 104 class C protein sequences. The two class C CID subclasses (class C1 and class C2) were readily distinguished, which ranged between 184 and 599, and between 92 and 312 amino acids residues in length, respectively. Three LOGO sequences were associated with both classes, LOGO #C1, which contained the CUE domain, and LOGO #C6 PAM2 and LOGO #C8 (Fig. 5). These results suggested that the classes are sequence related and that they may have a common origin. LOGO #C8 is an 11 residue sequence of unknown function that is present in all or almost all class C proteins (Additional file 7). CID5/IPD1 was previously described as a protein that encodes a CUE domain variant [24]. The CUE is a domain of about 50 amino acids that encode conserved motifs, which can interact with ubiquitin. An invariant proline residue and a di-leucine motif are highly conserved in the CUE domain (Fig. 6 and Additional file 8) [26]. CID5/IPD1 encoded a serine to the invariant proline. The results for the alignment of the CUE domain of class C CIDs indicated that most Brassicaceae of the C2 subclass and five monocots of the C1 subclass lack the P residue (single asterisks, Fig. 6 and Additional file 8). The leucine-leucine (LL) motif that can prevail in plants as methionine-leucine (ML) varied in only 2 of the 104 C1 subclass sequences (double asterisks, Additional file 8). Distinct LOGO sequences were also produced for each subclass. They included two LOGOs defined as previously identified domains DUF1771 and SMR in subclass C1 (Fig. 4); these two LOGOs were present in all of the class C1 sequences.

**Fig. 7:**
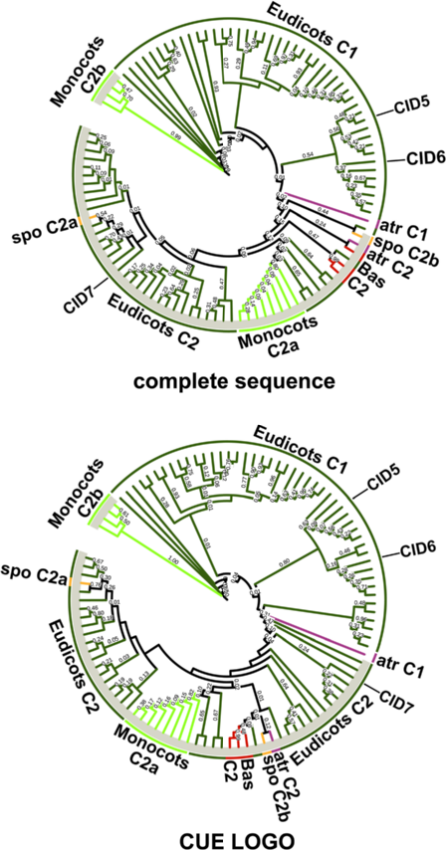
Phylogeny of CID C proteins. The CUE sequence LOGO (LOGO #C1) and the complete polypeptide sequence were used to obtain the trees; they were generated by the NJ method. CID C1 class is shadowed in gray. The color codes on the branches for groups of organisms are the same as in Fig. 4. Branch were named with the subclass code followed by a letter. The positions of the three *A. thaliana* paralogs, CID5, CID6 and CID7 are shown. The rectangular phylogeny is shown in Additional file 9. Trees generated using MP and ML are displayed in Additional file 10

The phylogenetic distribution of 104 class C CIDs was constructed using complete polypeptide sequences or the 41 amino acid segment comprising the CUE domain (LOGO #C1); trees were generated by the three methods NJ, ML and MP. With the NJ tree, except for *C. papaya* and *M. truncatula*, subclass C1 sequences clustered together on both trees (encircled in gray in Fig. 7 and Additional file 9); one major clade containing members of all species and in one minor clade four monocot sequences (see clade Monocots C2b). A similar clustering was observed when the ML and MP methods were used (see Additional file 10). In every tree, all of the monocot sequences were included in subclass C1 (light green branches in Fig. 7 and in Additional file 10). The two *S. polyrhiza* proteins clustered in this class even though they are probably unfinished sequences (mustard branches in Fig. 7 and in Additional file 10). The result that the two basal (*P. patens* and *S. moellendorffii*) and the monocot species were only present in subclass C1, suggested that subclass C2 arose from lack of retention of specific domains in eudicots, after gene duplication (at least the DUF1771 and SMR domains) (red and light green branches in Fig. 7 and in Additional file 10).

### Class D CIDs consists of highly conserved RNA-binding proteins

Class D includes six putative RNA-binding proteins (RBPs) (*CID8*, *CID9*, *CID10*, *CID11*, *CID12,* and *CID13*) that contain two RNA recognition motifs (RRMs). RBPs have central functions in RNA metabolism [13]. RRM is one of the various domains known to bind RNA. Similar to other eukaryotic organisms, genes in *A. thaliana* encode more than 200 RBPs; many of these are plant-specific [27, 28]. Except for some information regarding CID12, the functions of this group of CIDs are unknown. CID12 corresponds to RBP37, which may have a role in early embryogenesis and organ growth [29].

Class D orthologs, which encode two consecutive RRM domains, were identified in all species. The numbers varied from 2 in the basal species, *P. patens* and *S. moellendorffii*, to 10 in *G. raimondii* and 15 in *B. rapa*. Putative orthologs from this class were identified in 12 algae species. These orthologs were not included in our analysis because inspection of the sequences indicated that the PAM2 motif was not present (data not shown). It is possible that PAM2 was acquired in land plants; this hypothesis was not further investigated.

**Fig. 8:**
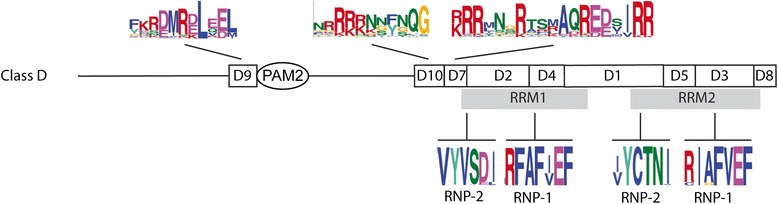
A broad view of the domain architecture of CID D proteins. The location of the two RRM domains is indicated below the sequence LOGOs that include them. The LOGOs of the RNP-2 and RNP-1 motifs for each RRM are shown. Relevant sequence LOGOs that are conserved in most of the sequences, LOGO #D9 LOGO #D10, and LOGO #D7 are displayed; the catalog of sequence LOGOs is displayed in Additional file 2

**Fig. 9:**
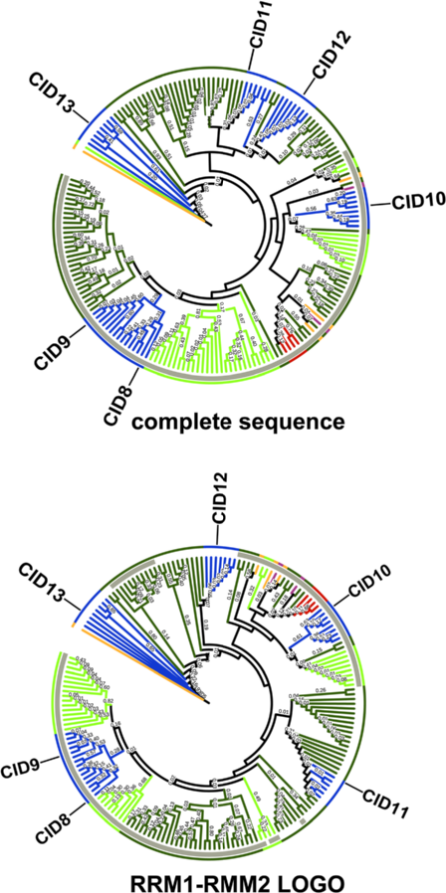
Phylogeny of CID D proteins. The sequence encompassing RRM1 and RRM2 motifs and the complete polypeptide sequence were used to obtain the trees by the NJ method. The positions of the six *A. thaliana* paralogs, CID8, CID9, CID10, CID11, CID12, and CID13 are shown. The color codes on the branches for groups of organisms are the same as in Fig. 4. The rectangular phylogeny is shown in Additional file 11

MEME searches were performed for a set of 184 class D protein sequences between 175 and 416 amino acids residues in length. Seven sequence LOGOs were found to extend the two RRM domains (LOGO #D7, LOGO #D2, LOGO #D4, LOGO #D1, LOGO #D5, LOGO #D3, and LOGO #D8; Fig. 9). The RRM is a widespread RNA-binding domain that includes two ribonucleoprotein motifs, RNP1 and RNP2 [27]. These two sequences are 6–8 amino acid residues motifs located to the middle, and close to the amino-terminal region, of the domain. The location and the primary sequence were conserved in almost all class D CID members (Fig. 8), which suggested that binding specificity is highly related in all members of the class. Sequence LOGOs common to almost all sequences were mapped adjacent to the RRM1 (LOGO #D10 and LOGO #D7) and the PAM2 (LOGO #D9) domains. The LOGO adjacent to the RRM1 was rich in arginine residues and LOGO #D9 in was rich in charged amino acid residues (Fig. 8). These results suggested that these LOGOs may have specific functions. We did not find evidence of major domain changes among members of class D that could be predicted using LOGO analysis. Although specific sequence LOGOs was generated for monocots and eudicots, their functional relevance remains unclear. Both RRM domains were maintained. This result suggests that these domains are involved in a highly conserved and specific function.

Construction of a phylogenetic distribution containing 184 CID D sequences was performed using the 174 amino acid segment comprising both RRM domains or the complete polypeptide sequences. Since subclasses were not inferred from the LOGO analysis of the pCID D sequences, we only constructed a tree by the NJ method (Fig. 9 and Additional file 11). The tree based on complete sequences had a major clade that contained all members of the two basal species, almost all monocots, and the members of most of the eudicot species analyzed. This clade contained three of the *A. thaliana* paralogs, CID8, CID9, and CID10. Consistencies were detected when the corresponding clade in the tree based on RRMs were compared. For instance, CID8 and CID9 were grouped in the same clade, suggesting that this is the most recent duplication of CID C genes. Likewise, CID10 grouped with the basal species in both trees indicating that it may be the earlier gene copy of this class (red branch in Fig. 9).

## Discussion

Diversification in regulatory programs is an indication of the ability of an organism to respond to multiple developmental and environmental cues. The evolution of specialized regulatory mechanisms endows organisms with an exceptional assortment of regulatory programs that fine-tune gene regulation at several levels [30–32]. As sessile organisms, plants have evolved elaborate and redundant regulatory mechanisms that fine-tune growth and development in response to the environment [33, 34]. The posttranscriptional level of gene expression is an important step in regulation. Precise interaction among regulators within assemblies can control the outcomes of specific transcripts (e.g., half-life stability and the ability to be translated). PABP is a conserved eukaryotic protein that interacts with the poly(A) tail of messenger RNAs having roles in mRNA stability and translation control [35]. One to a few PABPs genes are found in fungi and animals, but a significant expansion in the gene number is evident throughout plant evolutionary history [3]. In this work, we performed a phylogenetic analysis of three classes of CIDs, the interaction partners of the MLLE domain of PABPs [13]. We found that variants that arose during evolution occurred frequently among CID classes. Based on the fact that CIDs may participate in PABPs assemblies, CID subclasses may result in paralog interference [31]. Although this interference is predictably unfavorable, it is often bypassed or resolved during evolution. The fact that CIDs subclasses are maintained since early lineages suggests their association within PABP assemblies. These results reinforce the idea that assorted control mechanisms evolved in plants to allow them to adjust to a changing environment. Yet, at this point, with the absence of proper experimental evidence we ought also to consider these variants to be selectively neutral, exerting independent functions. One exception, Class D, consists of highly conserved RNA-binding proteins. In our analysis of this class, we did not detect any sequence variations that might suggest divergence. More than one copy of CID D proteins was present in the analyzed plants. This result suggested that they are redundant proteins that may have very similar roles.

Our previous findings for class B CIDs suggested that functional specialization is present in ataxin-2 orthologs. A subclass was found that might have novel subcellular localization and functional properties; it lacks the LsmAD domain that includes a clathrin-mediated trans-Golgi signal and participates in the recruitment of the helicase DDX6 [18]. Similarly, CID class A and C variants were also identified. Functional diversity has been predicted for CID A. CID1/ERD15 functions in diverse stress pathways. The *G. max* ortholog, GmERD15, is a transcription factor that binds to the NRP-B promoter and functions as an early component that links endoplasmic reticulum stress to an osmotic stress-induced cell death signal [23]. The results of a a transactivation experiment in yeast revealed that CID1/ERD15 and GmERD15 work in different ways. GmERD15 displays transactivation activity, but CID1/ERD15 does not; CID1/ERD15 also does not bind to the NRP-B promoter in yeast [22, 23]. These results suggest that functional divergence of these two orthologs has occurred. Whether CID1/ERD15 is a transcription factor needs to be evaluated. The results of our a previous assay suggested that CID1/ERD15 encodes an activation domain that is functional in yeast [13]. It is possible that CID A proteins are transcription factors consisting of paralogs that diverged during evolution.

The results of the domain architecture and the phylogenetic distribution analyses supported the hypothesis that functional divergence occurred among class CID A genes. A specific LOGO generated on putative GmERD15 orthologs (LOGO #A6, Fig. 4) of all species except the basal species, *P. patens* and *S. moellendorffii*, and the six Brassicaceae that were analyzed. The result that sequence LOGOs did not generate in the two basal species indicates that specific domains were acquired throughout evolution. Moreover, orthologs containing LOGO #A6 clustered in both phylogenetic trees based on complete sequences or in the PAE1 LOGO. These clusters did not include any of the Brassicaceae species analyzed (Fig. 5). Since *P. patens* and *S. moellendorffii* sequences are basal to the GmERD15 orthologs and the Brassicaceae species are not the same lineage, it is possible that this specific ortholog was lost early in the Brassicaceae lineage.

Variants of class C CIDs were readily identified. Class C1 variants are present in both monocot and eudicot species. Class B variants are present only in eudicots. Class C1 encodes larger proteins than class C2 and contains domains that are not present in C2 (Fig. 6). Class C2 likely underwent rearrangements, which included deletions, early during the split between monocots and eudicots. At least two regions are common to both classes, CUE and LOGO #C8. CUE is an evolutionarily conserved domain that binds directly to monoubiquitin and participates in ubiquitination [26]. CID5/IPD1 (class 2) is involved in a light-dependent endoreduplication pathway, controlling the endocycle during hypocotyl elongation. CID5/IPD1 is described as a protein containing a CUE domain variant because a highly conserved amino acid residue within the CUE domain involved in ubiquitin binding is missing; a proline residue has been replaced by a serine. The CUE domain is likely diverging into distinct lineages [24]. Our results suggested that this divergence specifically occurred in the class C2 proteins, from the six Brassicaceae examined (Fig. 7). Each of these Brassicaceae members have at least two copies of class C2 genes. One of the copies still encodes the highly conserved proline residue only in *A. thaliana* and *T. halophila*. Similarly, major sequence variation is present in four monocot sequences from class C1, which is consistent with their grouping in a separate branch in the phylogenetic tree (Fig. 7 and Fig. 8). Because this proline residue is essential for monoubiquitin binding, whether the CUE domain in these proteins remains as a component of the ubiquitination machinery remains to be determined. The function of the SMR domain encoded in class C1 that is a widespread in eukaryotes continues to be an enigma [25].

## Conclusions

Functional analysis of CID proteins is in its infancy in plants. Because there is obvious diversification among various classes of CIDs, the functional analysis will be helpful to determine whether diversification is neutral or whether it is important for plant adaptation to the environment, or for the acquisition of specific traits during evolution. Association of CIDs within PABPs assemblies could be envisioned based on the presence of the PAM2 domain and on the fact that PABPs interact with many other proteins involved in the regulation of protein synthesis or mRNA metabolism. PABPs assemblies are basic to every facet of the biology of a plant, so CIDs are likely to have important roles in the regulation of growth, development, and environmental responses. Since the loss of domains often results in competitive interference between duplicates [31, 32, 36], functional analysis will also be imperative to establish the hook up of CID subclasses to the assemblies.

## Additional files

Additional file 1:
**Retrieved Viridiplantae CID genes.** (XLSX 20 kb) Additional file 2:
**Catalog of sequence LOGOs generated from CID classes A, C, and D.** (PDF 1894 kb) Additional file 3:
**Alignment of the CID A1 proteins.** Domain architecture is represented at the top, including PAM2 and GmERD15-TFD, and the numbers of the sequence LOGOs that mapped to CID A2 proteins. The primary sequence for each LOGO is shown. Sequence alignment was obtained using ClustalX 2.0.12 software, and a default color code was applied. The locations of regions encompassing LOGO #A5, LOGO #A7, and LOGO #A10 are enclosed by rectangles. (PDF 6840 kb) Additional file 4:
**Alignment of the CID A2 proteins.** Domain architecture is represented at the top, including PAM2 and GmERD15-TFD, and sequence LOGOs mapped to CID A2 proteins, denoted by number. Sequence alignment was obtained using ClustalX 2.0.12, and a default color code was applied. The locations of regions encompassing LOGO #A22 and LOGO #A6 (GmERD15-TFD) are enclosed by rectangles. (PDF 4775 kb) Additional file 5:
**Rectangular phylogenetic tree of class A CIDs proteins based on the PAE1 LOGO (LOGO #A1), or on the complete polypeptide sequence.** The topology was generated using the NJ method. Species and gene names are those presented in Additional file 1. The color codes on the branches for groups of organisms are the same as in Fig. 4; protein names are displayed. (PDF 4320 kb) Additional file 6:
**Circular phylogenetic tree of class A CIDs proteins based on the PAE1 LOGO (LOGO #A1), or on the complete polypeptide sequence.** The topology was generated using the maximum-parsimony (MP) or the maximum-likelihood (ML), as indicated (see Methods). (*) are in dissimilar places in both MP trees. Color codes of branches are as depicted in Fig. 4. Labels on branches are based on the NJ trees from Fig. 4. (PDF 18177 kb) Additional file 7:
**Protein sequence alignment for class C sequence LOGO #C8.** The sequence alignments were performed using ClustalX 2.0.12; default colors were used. (PDF 14808 kb) Additional file 8:
**Alignment of the CUE motif of CID C proteins.** The two CID C classes and the Brassicaceae species are indicated by brackets. The region encompassing sequence LOGO #1 from all CID C sequences was aligned using ClustalX 2.0.12 software, and a default color code was applied. The locations of important residues are highlighted by arrows (the invariant proline residue and a di-leucine motif). (*) indicates substitution of the invariant proline residue, (**) indicates substitution at the di-leucine motif. (PDF 4954 kb) Additional file 9:
**Rectangular phylogenetic tree of class C CIDs proteins based on the CUE motif (LOGO #C1), or on the complete polypeptide sequence.** The topology was generated using the neighbor-joining (NJ) method. Species and gene names are those presented in Additional file 1. The color codes on the branches for groups of organisms are the same as in Fig. 4; protein names are displayed. (PDF 16124 kb) Additional file 10:
**Circular phylogenetic tree of class C CIDs proteins based on the CUE motif (LOGO #C1), or on the complete polypeptide sequence.** The topology was generated using the MP or the ML methods, as indicated (see Methods). Color codes of branches are as depicted in Fig. 4. Labels of branches are based on the NJ trees from Fig. 7. (PDF 9581 kb) Additional file 11:
**Rectangular phylogenetic tree of class D CIDs proteins based on RRM1 and RRM2 motifs, or on the complete polypeptide sequence, as described in Fig.** **9** **.** (PDF 27070 kb)

## References

[1] Wells SE, Hillner PE, Vale RD, Sachs AB (1998). Circularization of mRNA by eukaryotic translation initiation factors. Mol Cell.

[2] Belostotsky DA (2003). Unexpected complexity of poly (A)-binding protein gene families in flowering plants: three conserved lineages that are at least 200 million years old and possible auto-and cross-regulation. Genetics.

[3] Gallie DR, Liu R (2014). Phylogenetic analysis reveals dynamic evolution of the poly (A)-binding protein gene family in plants. BMC Evol Biol.

[4] Mangus DA, Evans MC, Jacobson A (2003). Poly (A)-binding proteins: multifunctional scaffolds for the post-transcriptional control of gene expression. Genome Biol.

[5] Kozlov G, M√©nade M, Rosenauer A, Nguyen L, Gehring K (2010). Molecular determinants of PAM2 recognition by the MLLE domain of poly (A)-binding protein. J Mol Biol.

[6] Eliseeva I, Lyabin D, Ovchinnikov L (2013). Poly (A)-binding proteins: structure, domain organization, and activity regulation. Biochemistry (Moscow).

[7] Kessler SH, Sachs AB (1998). RNA recognition motif 2 of yeast Pab1p is required for its functional interaction with eukaryotic translation initiation factor 4G. Mol Cell Biol.

[8] Gingras A-C, Raught B, Sonenberg N (1999). eIF4 initiation factors: effectors of mRNA recruitment to ribosomes and regulators of translation. Annu Rev Biochem.

[9] Safaee N, Kozlov G, Noronha AM, Xie J, Wilds CJ, Gehring K (2012). Interdomain allostery promotes assembly of the poly (A) mRNA complex with PABP and eIF4G. Mol Cell.

[10] Derry M, Yanagiya A, Martineau Y, Sonenberg N (2006). Regulation of poly (A)-binding protein through PABP-interacting proteins. Cold Spring Harbor symposia on quantitative biology.

[11] Albrecht M, Lengauer T (2004). Survey on the PABC recognition motif PAM2. Biochem Biophys Res Commun.

[12] Xie J, Kozlov G, Gehring K (2014). The “tale” of poly (A) binding protein: the MLLE domain and PAM2-containing proteins. Biochim Biophys Acta.

[13] Bravo J, Aguilar-Henonin L, Olmedo G, Guzman P (2005). Four distinct classes of proteins as interaction partners of the PABC domain of Arabidopsis thaliana Poly (A)-binding proteins. Mol Genet Genomics.

[14] Larkin MA, Blackshields G, Brown N, Chenna R, McGettigan PA, McWilliam H (2007). Clustal W and Clustal X version 2.0. Bioinformatics.

[15] Tamura K, Stecher G, Peterson D, Filipski A, Kumar S (2013). MEGA6: molecular evolutionary genetics analysis version 6.0. Mol Biol Evol.

[16] Letunic I, Bork P (2007). Interactive Tree Of Life (iTOL): an online tool for phylogenetic tree display and annotation. Bioinformatics.

[17] Bailey TL, Johnson J, Grant CE, Noble WS. The MEME suite. Nucleic Acids Res. 2015; 43(W1):W39-49. 10.1093/nar/gkv416 PMC4489269 25953851

[18] Jiménez-López D, Guzmán P (2014). Insights into the evolution and domain structure of ataxin-2 proteins across eukaryotes. BMC Res Notes.

[19] Kiyosue T, Yamaguchi-Shinozaki K, Shinozaki K (1994). ERD15, a cDNA for a dehydration-induced gene from Arabidopsis thaliana. Plant Physiol.

[20] Kariola T, Brader G, Helenius E, Li J, Heino P, Palva ET (2006). Early responsive to dehydration 15, a negative regulator of abscisic acid responses in Arabidopsis. Plant Physiol.

[21] Aalto MK, Helenius E, Kariola T, Pennanen V, Heino P, Horak H (2012). ERD15-An attenuator of plant ABA responses and stomatal aperture. Plant Sci.

[22] Alves MS, Fontes EP, Fietto LG (2011). Early responsive to dehydration 15, a new transcription factor that integrates stress signaling pathways. Plant Signal Behav.

[23] Alves MS, Reis PA, Dadalto SP, Faria JA, Fontes EP, Fietto LG (2011). A novel transcription factor, ERD15 (Early Responsive to Dehydration 15), connects endoplasmic reticulum stress with an osmotic stress-induced cell death signal. J Biol Chem.

[24] Tsumoto Y, Yoshizumi T, Kuroda H, Kawashima M, Ichikawa T, Nakazawa M (2006). Light-dependent polyploidy control by a CUE protein variant in Arabidopsis. Plant Mol Biol.

[25] Liu S, Melonek J, Boykin LM, Small I, Howell KA (2013). PPR-SMRs: ancient proteins with enigmatic functions. RNA Biol.

[26] Shih SC, Prag G, Francis SA, Sutanto MA, Hurley JH, Hicke L (2003). A ubiquitin-binding motif required for intramolecular monoubiquitylation, the CUE domain. EMBO J.

[27] Cléry A, Blatter M, Allain FH (2008). RNA recognition motifs: boring? Not quite. Curr Opin Struct Biol.

[28] Lorkovic ZJ, Barta A (2002). Genome analysis: RNA recognition motif (RRM) and K homology (KH) domain RNA-binding proteins from the flowering plant Arabidopsis thaliana. Nucleic Acids Res.

[29] Hecht V, Stiefel V, Delseny M, Gallois P (1997). A new Arabidopsis nucleic-acid-binding protein gene is highly expressed in dividing cells during development. Plant Mol Biol.

[30] Teichmann SA, Babu MM (2004). Gene regulatory network growth by duplication. Nat Genet.

[31] Baker CR, Hanson-Smith V, Johnson AD (2013). Following gene duplication, paralog interference constrains transcriptional circuit evolution. Science.

[32] Finnigan GC, Hanson-Smith V, Stevens TH, Thornton JW (2012). Evolution of increased complexity in a molecular machine. Nature.

[33] Maere S, De Bodt S, Raes J, Casneuf T, Van Montagu M, Kuiper M (2005). Modeling gene and genome duplications in eukaryotes. Proc Natl Acad Sci U S A.

[34] Less H, Angelovici R, Tzin V, Galili G (2011). Coordinated gene networks regulating Arabidopsis plant metabolism in response to various stresses and nutritional cues. Plant Cell.

[35] Mata J, Marguerat S, Bähler J (2005). Post-transcriptional control of gene expression: a genome-wide perspective. Trends Biochem Sci.

[36] Bridgham JT, Brown JE, Rodríguez-Marí A, Catchen JM, Thornton JW (2008). Evolution of a new function by degenerative mutation in cephalochordate steroid receptors. PLoS Genet.

[37] Federhen S (2012). The NCBI taxonomy database. Nucleic Acids Res.

