# Supplementary material for: Evolutionary history exposes radical diversification among classes of interaction partners of the MLLE domain of plant poly(A)-binding proteins
Source: BMC Evol Biol. 2015 Sep 16;15:195. doi: 10.1186/s12862-015-0475-1 (PMC4574140; doi:10.1186/s12862-015-0475-1)

Class A

Logo Number **Sequence**

General

[A1] 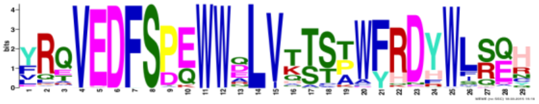 PAE1

[A2] 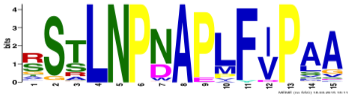 PAM2

[A3] 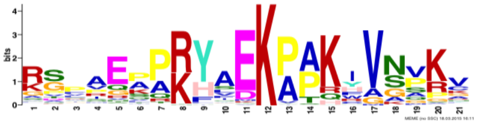

[A4] 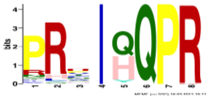

Subclass A1

[A5] 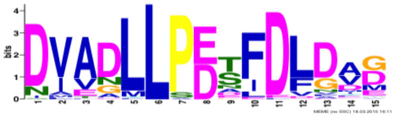

[A6] 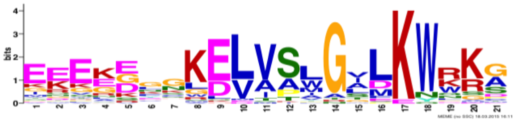

[A8] 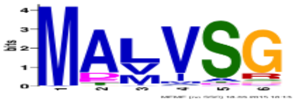

[A9] 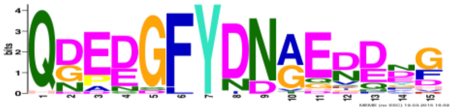

[A10] 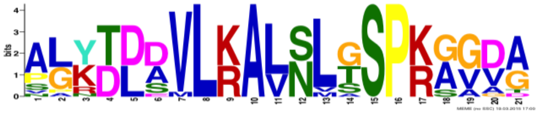

[A12] 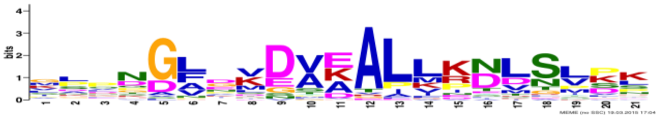

[A15] 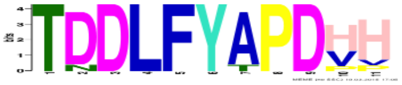

[A22] 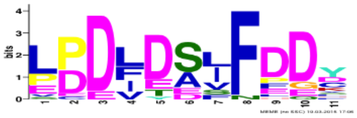

Subclass A2

[A7]

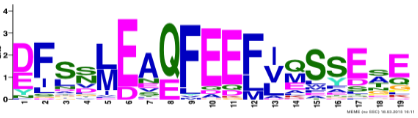

[A14]

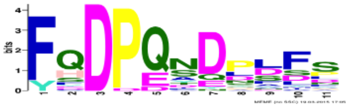

[A16]

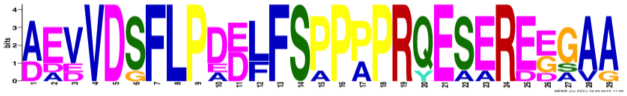

Class C

General

[C1]

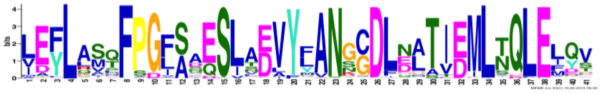

CUE

[C6]

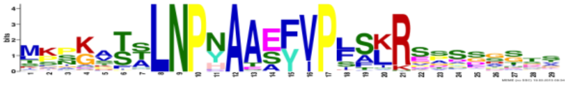

PAM2

[C8]

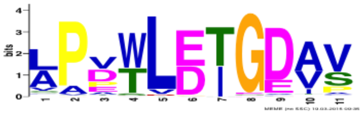

Subclass C1

[C2]

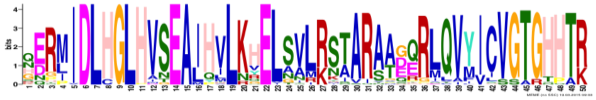

[C3]

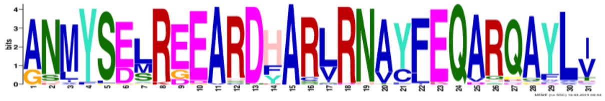

[C4]

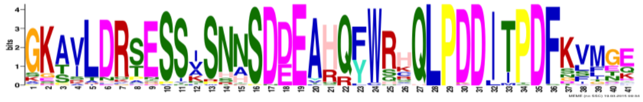

[C5]

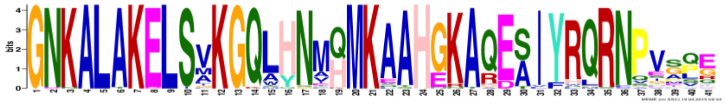

[C7]

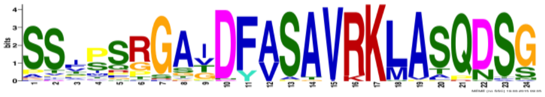

[C9]

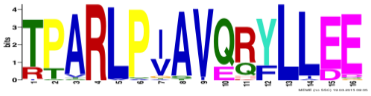

[C10]

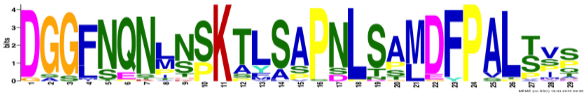

[C11]

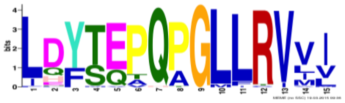

[C12]

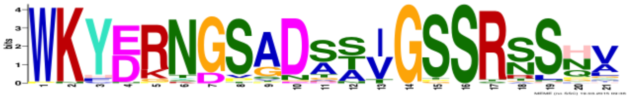

[C13]

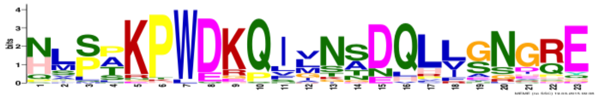

[C14]

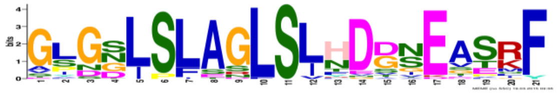

[C15]

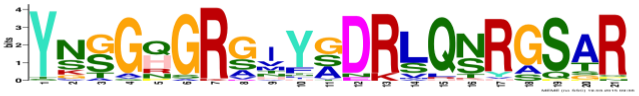

[C16]

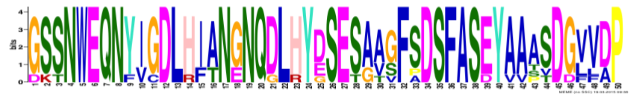

[C17]

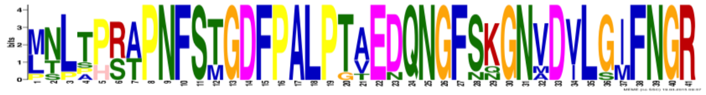

[C18]

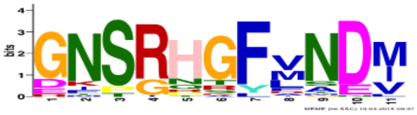

[C19]

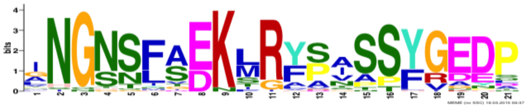

[C20]

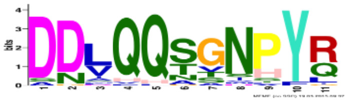

[C23]

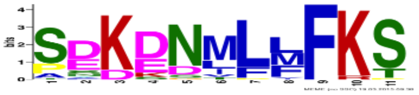

[C24]

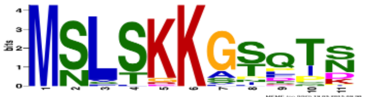

[C25]

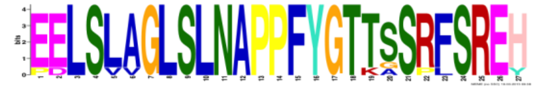

[C27]

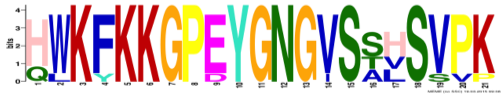

[C28]

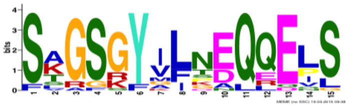

[C29]

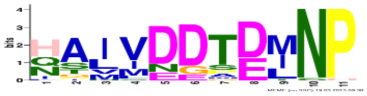

[C30]

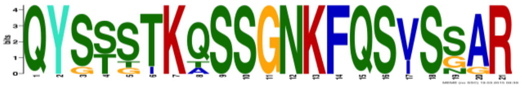

Subclass C2

[C21]

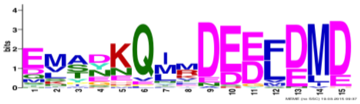

[C22]

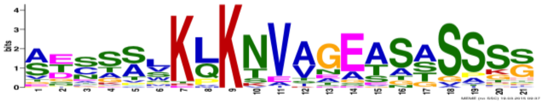

[C26]

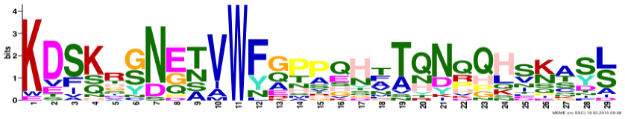

Class D

[D1]

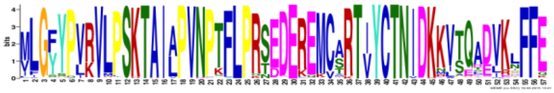

RRM1

[D2]

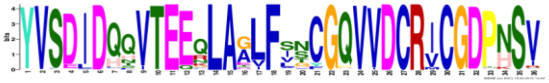

RRM2

[D3]

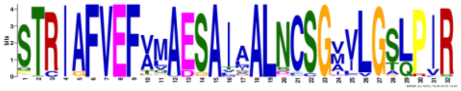

[D4]

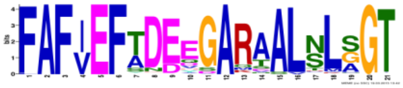

[D5]

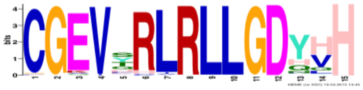

[D6]

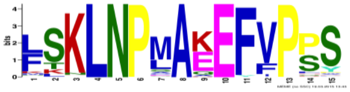

PAM2

[D7]

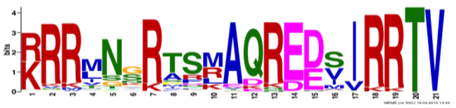

[D8]

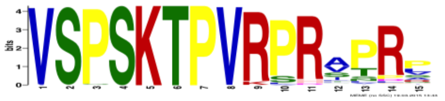

[D9]

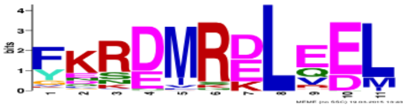

[D10]

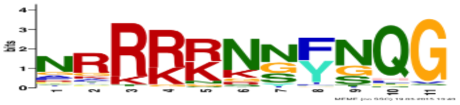

[D11]

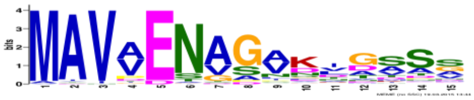

[D13]

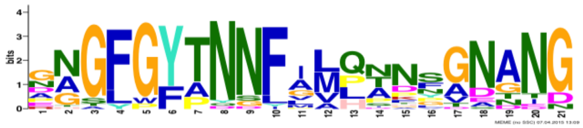

[D14]

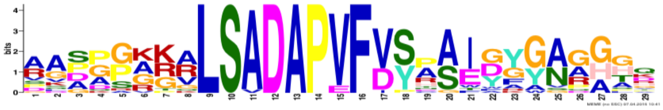

[D15]

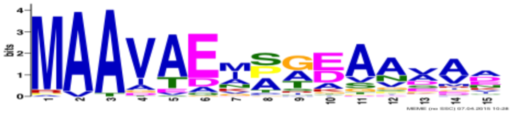

Supplement: Additional file 2: — Catalog of sequence LOGOs generated from CID classes A, C, and D. (PDF 1894 kb) [file 12862_2015_475_MOESM2_ESM.pdf]
