# Supplementary material for: Evolutionary history exposes radical diversification among classes of interaction partners of the MLLE domain of plant poly(A)-binding proteins
Source: BMC Evol Biol. 2015 Sep 16;15:195. doi: 10.1186/s12862-015-0475-1 (PMC4574140; doi:10.1186/s12862-015-0475-1)
Supplement: Additional file 3: — Alignment of the CID A1 proteins. Domain architecture is represented at the top, including PAM2 and GmERD15-TFD, and the numbers of the sequence LOGOs that mapped to CID A2 proteins. The primary sequence for each LOGO is shown. Sequence alignment was obtained using ClustalX 2.0.12 software, and a default color code was applied. The locations of regions encompassing LOGO #A5, LOGO #A7, and LOGO #A10 are enclosed by rectangles. (PDF 6840 kb) [file 12862_2015_475_MOESM3_ESM.pdf]

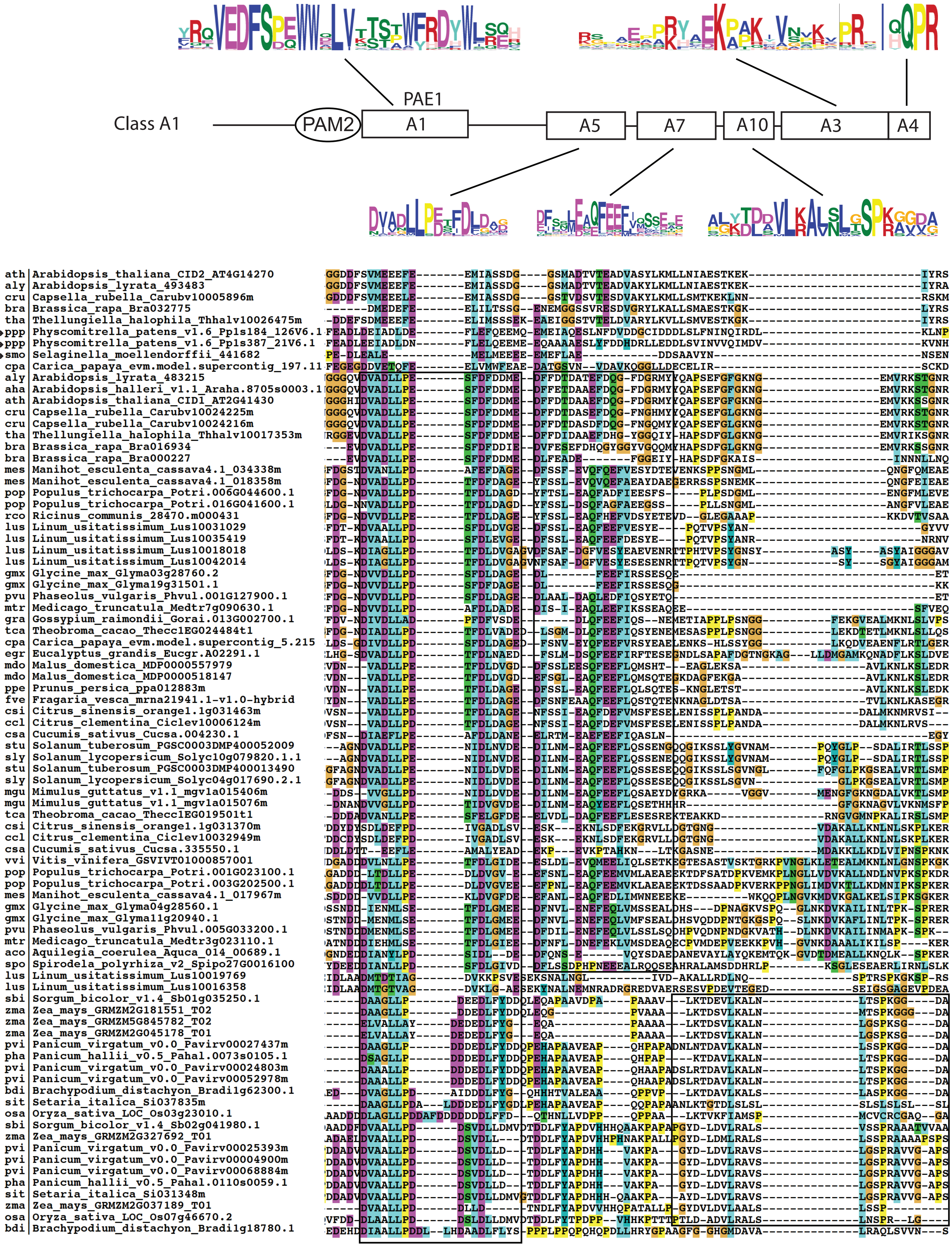

PAE1
Class A1
PAM2
A1
A5
A7
A10
A3
A4
ath Arabidopsis_thaliana_CID2_AT4G14270
aly Arabidopsis_lyrata_493483
cru Capsella_rubella_Carubv10005896m
bra Brassica_rapa_Bra032775
tha Thellungiella_halophila_Thhalv10026475m
ppp Physcomitrella_patens_v1.6_Pp1s184_126V6.1
ppp Physcomitrella_patens_v1.6_Pp1s387_21V6.1
smo Selaginella_moellendorffii_441682
cpa Carica_papaya_evm.model.supercontig_197.11
aly Arabidopsis_lyrata_483215
aha Arabidopsis_halleri_v1.1_Araha.8705s0003.1
ath Arabidopsis_thaliana_CID1_AT2G41430
cru Capsella_rubella_Carubv10024225m
cru Capsella_rubella_Carubv10024216m
tha Thellungiella_halophila_Thhalv10017353m
bra Brassica_rapa_Bra016934
bra Brassica_rapa_Bra000227
mes Manihot_esculenta_cassava4.1_034338m
mes Manihot_esculenta_cassava4.1_018358m
pop Populus_trichocarpa_Potri.006G044600.1
pop Populus_trichocarpa_Potri.016G041600.1
rco Ricinus_communis_28470.m000431
lus Linum_usitatissimum_Lus10031029
lus Linum_usitatissimum_Lus10035419
lus Linum_usitatissimum_Lus10018018
lus Linum_usitatissimum_Lus10042014
gmx Glycine_max_Glyma03g28760.2
gmx Glycine_max_Glyma19g31501.1
pvu Phaseolus_vulgaris_Phvul.001G127900.1
mtr Medicago_truncatula_Medtr7g090630.1
gra Gossypium_raimondii_Gorai.013G002700.1
tca Theobroma_cacao_Thecc1EG024484t1
cpa Carica_papaya_evm.model.supercontig_5.215
egr Eucalyptus_grandis_Eucgr.A02291.1
mdo Malus_domestica_MDP0000557979
mdo Malus_domestica_MDP0000518147
ppe Prunus_persica_ppa012883m
fve Fragaria_vesca_mrna21941.1-v1.0-hybrid
csi Citrus_sinensis_orange1.1g031463m
ccl Citrus_clementina_Ciclev10006124m
csa Cucumis_sativus_Cucsa.004230.1
stu Solanum_tuberosum_PGSC0003DMP400052009
sly Solanum_lycopersicum_Solyc10g079820.1.1
stu Solanum_tuberosum_PGSC0003DMP400013490
sly Solanum_lycopersicum_Solyc04g017690.2.1
mgu Mimulus_guttatus_v1.1_mgv1a015406m
mgu Mimulus_guttatus_v1.1_mgv1a015076m
tca Theobroma_cacao_Thecc1EG019501t1
csi Citrus_sinensis_orange1.1g031370m
ccl Citrus_clementina_Ciclev10032949m
csa Cucumis_sativus_Cucsa.335550.1
vvi Vitis_vinifera_GSVIVT01000857001
pop Populus_trichocarpa_Potri.001G023100.1
pop Populus_trichocarpa_Potri.003G202500.1
mes Manihot_esculenta_cassava4.1_017967m
gmx Glycine_max_Glyma04g28560.1
gmx Glycine_max_Glyma11g20940.1
pvu Phaseolus_vulgaris_Phvul.005G033200.1
mtr Medicago_truncatula_Medtr3g023110.1
aco Aquilegia_coerulea_Aquca_014_00689.1
spo Spirodela_polyrhiza_v2_Spipo27G0016100
lus Linum_usitatissimum_Lus10019769
lus Linum_usitatissimum_Lus10016358
sbi Sorgum_bicolor_v1.4_Sb01g035250.1
zma Zea_mays_GRMZM2G181551_T02
zma Zea_mays_GRMZM5G845782_T02
zma Zea_mays_GRMZM2G045178_T01
pvi Panicum_virgatum_v0.0_Pavirv00027437m
pha Panicum_hallii_v0.5_Pahal.0073s0105.1
pvi Panicum_virgatum_v0.0_Pavirv00024803m
pvi Panicum_virgatum_v0.0_Pavirv00052978m
bdi Brachypodium_distachyon_Bradi1g62300.1
sit Setaria_italica_Si037835m
osa Oryza_sativa_LOC_Os03g23010.1
sbi Sorgum_bicolor_v1.4_Sb02g041980.1
zma Zea_mays_GRMZM2G327692_T01
pvi Panicum_virgatum_v0.0_Pavirv00025393m
pvi Panicum_virgatum_v0.0_Pavirv00004900m
pvi Panicum_virgatum_v0.0_Pavirv00068884m
pha Panicum_hallii_v0.5_Pahal.0110s0059.1
sit Setaria_italica_Si031348m
zma Zea_mays_GRMZM2G037189_T01
osa Oryza_sativa_LOC_Os07g46670.2
bdi Brachypodium_distachyon_Bradi1g18780.1
