# Supplementary material for: Evolutionary history exposes radical diversification among classes of interaction partners of the MLLE domain of plant poly(A)-binding proteins
Source: BMC Evol Biol. 2015 Sep 16;15:195. doi: 10.1186/s12862-015-0475-1 (PMC4574140; doi:10.1186/s12862-015-0475-1)
Supplement: Additional file 4: — Alignment of the CID A2 proteins. Domain architecture is represented at the top, including PAM2 and GmERD15-TFD, and sequence LOGOs mapped to CID A2 proteins, denoted by number. Sequence alignment was obtained using ClustalX 2.0.12, and a default color code was applied. The locations of regions encompassing LOGO #A22 and LOGO #A6 (GmERD15-TFD) are enclosed by rectangles. (PDF 4775 kb) [file 12862_2015_475_MOESM4_ESM.pdf]

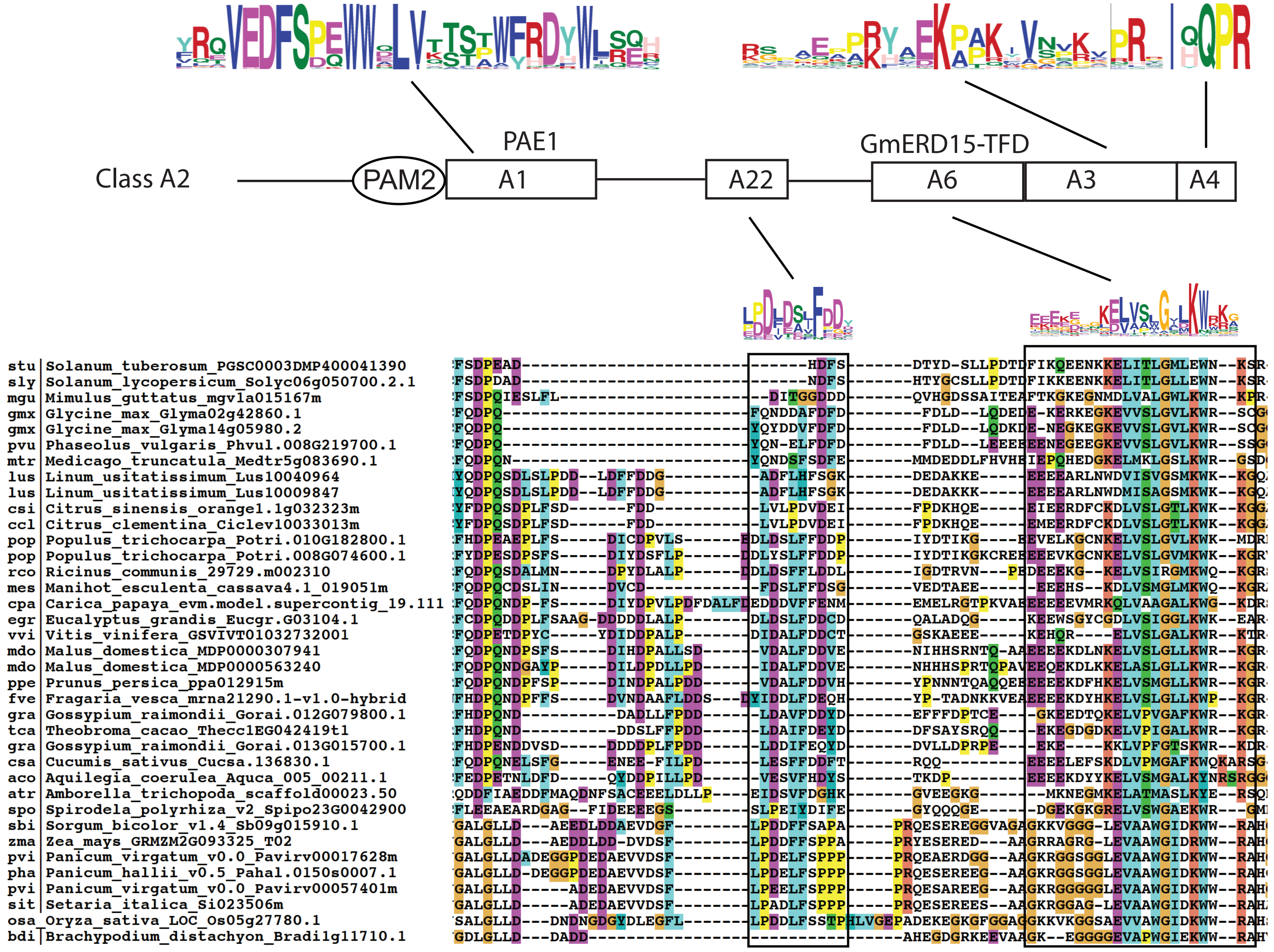

PAE1
GmERD15-TFD
Class A2
PAM2
A1
A22
A6
A3
A4
stu Solanum_tuberosum_PGSC0003DMP400041390
sly Solanum_lycopersicum_Solyc06g050700.2.1
mgu Mimulus_guttatus_mgv1a015167m
gmx Glycine_max_Glyma02g42860.1
gmx Glycine_max_Glyma14g05980.2
pvu Phaseolus_vulgaris_Phvul.008G219700.1
mtr Medicago_truncatula_Medtr5g083690.1
lus Linum_usitatissimum_Lus10040964
lus Linum_usitatissimum_Lus10009847
csi Citrus_sinensis_orange1.1g032323m
ccl Citrus_clementina_Ciclev10033013m
pop Populus_trichocarpa_Potri.010G182800.1
pop Populus_trichocarpa_Potri.008G074600.1
rco Ricinus_communis_29729.m002310
mes Manihot_esculenta_cassava4.1_019051m
cpa Carica_papaya_evm.model.supercontig_19.111
egr Eucalyptus_grandis_Eucgr.G03104.1
vvi Vitis_vinifera_GSVIVT01032732001
mdo Malus_domestica_MDP0000307941
mdo Malus_domestica_MDP0000563240
ppe Prunus_persica_ppa012915m
fve Fragaria_vesca_mrna21290.1-v1.0-hybrid
gra Gossypium_raimondii_Gorai.012G079800.1
tca Theobroma_cacao_Thecc1EG042419t1
gra Gossypium_raimondii_Gorai.013G015700.1
csa Cucumis_sativus_Cucsa.136830.1
aco Aquilegia_coerulea_Aquca_005_00211.1
atr Amborella_trichopoda_scaffold00023.50
spo Spirodela_polyrhiza_v2_Spipo23G0042900
sbi Sorgum_bicolor_v1.4_Sb09g015910.1
zma Zea_mays_GRMZM2G093325_T02
pvi Panicum_virgatum_v0.0_Pavirv00017628m
pha Panicum_hallii_v0.5_Pahal.0150s0007.1
pvi Panicum_virgatum_v0.0_Pavirv00057401m
sit Setaria_italica_Si023506m
osa Oryza_sativa_LOC_Os05g27780.1
bdi Brachypodium_distachyon_Bradi1g11710.1
