# Supplementary figures and images for: Evolutionary history exposes radical diversification among classes of interaction partners of the MLLE domain of plant poly(A)-binding proteins
Source: BMC Evol Biol. 2015 Sep 16;15:195. doi: 10.1186/s12862-015-0475-1 (PMC4574140; doi:10.1186/s12862-015-0475-1)

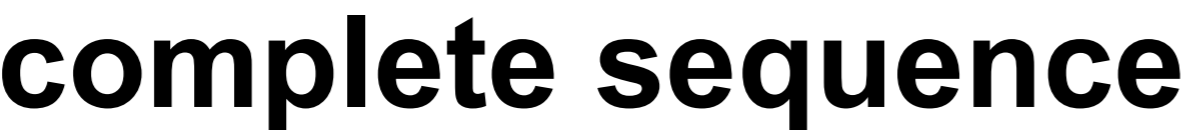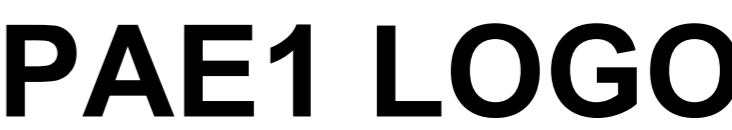

Supplement: Additional file 5: — Rectangular phylogenetic tree of class A CIDs proteins based on the PAE1 LOGO (LOGO #A1), or on the complete polypeptide sequence. The topology was generated using the NJ method. Species and gene names are those presented in Additional file 1. The color codes on the branches for groups of organisms are the same as in Fig. 4; protein names are displayed. (PDF 4320 kb) [file 12862_2015_475_MOESM5_ESM.pdf]

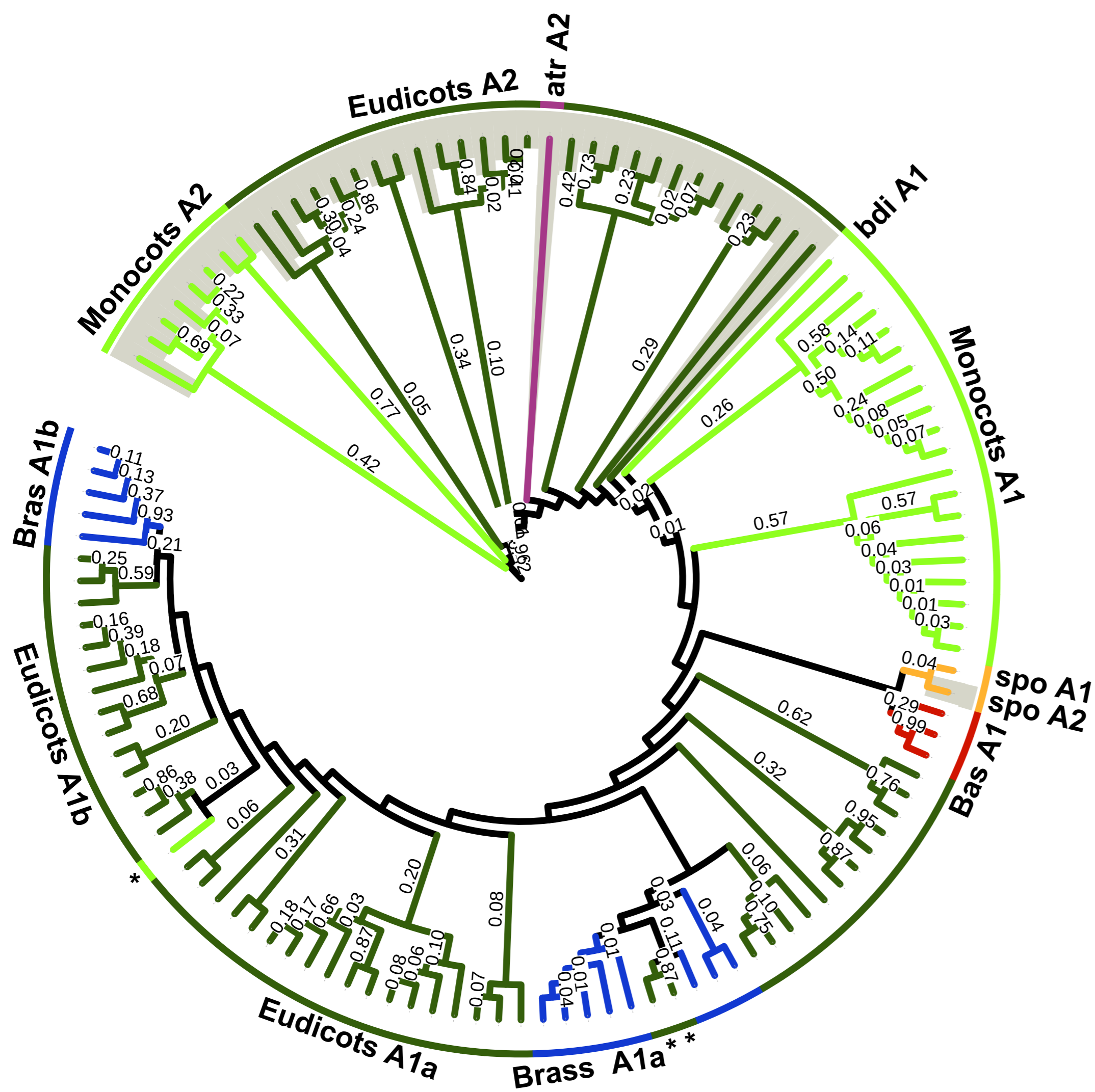

complete sequence MP

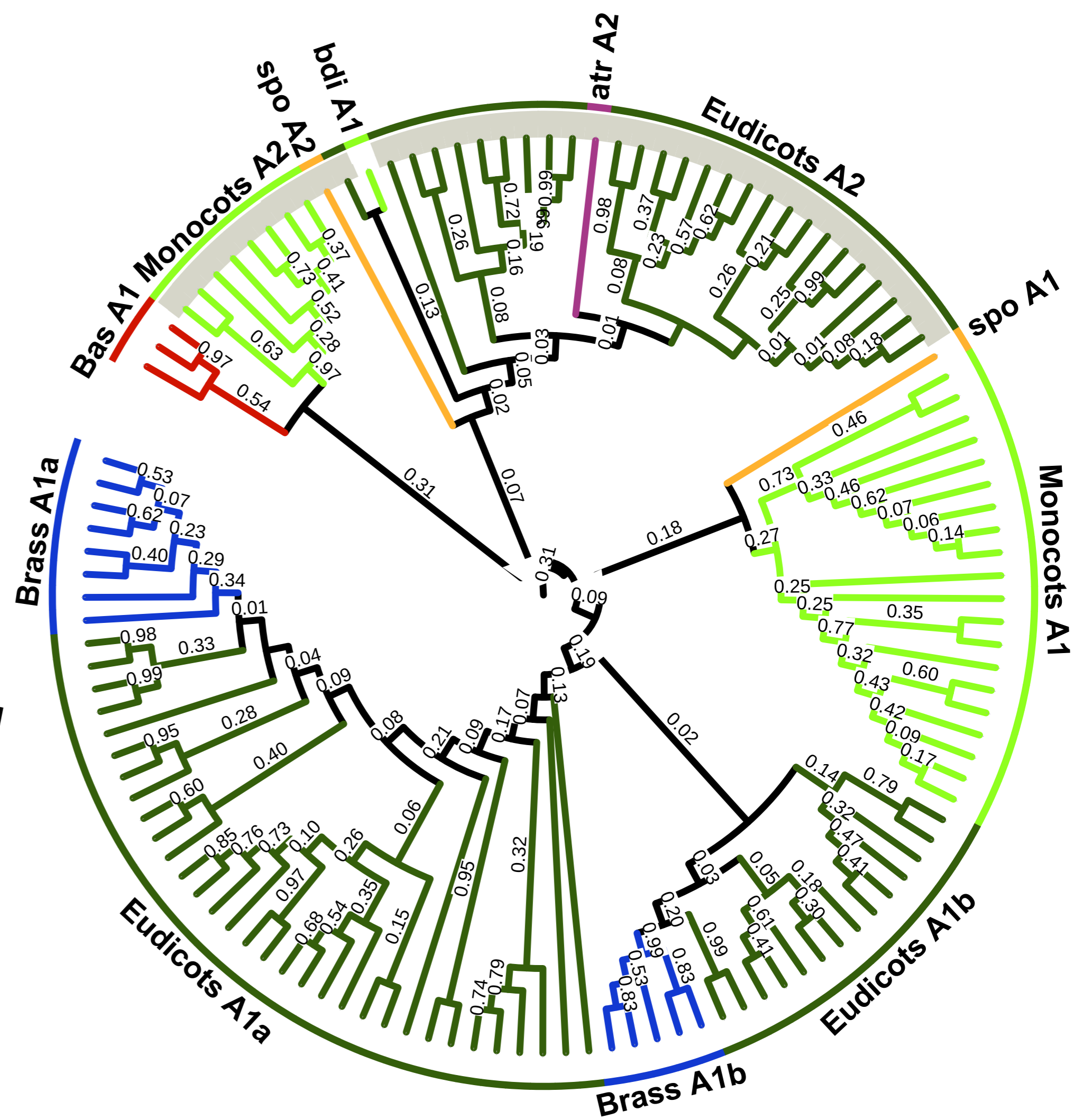

complete sequence ML

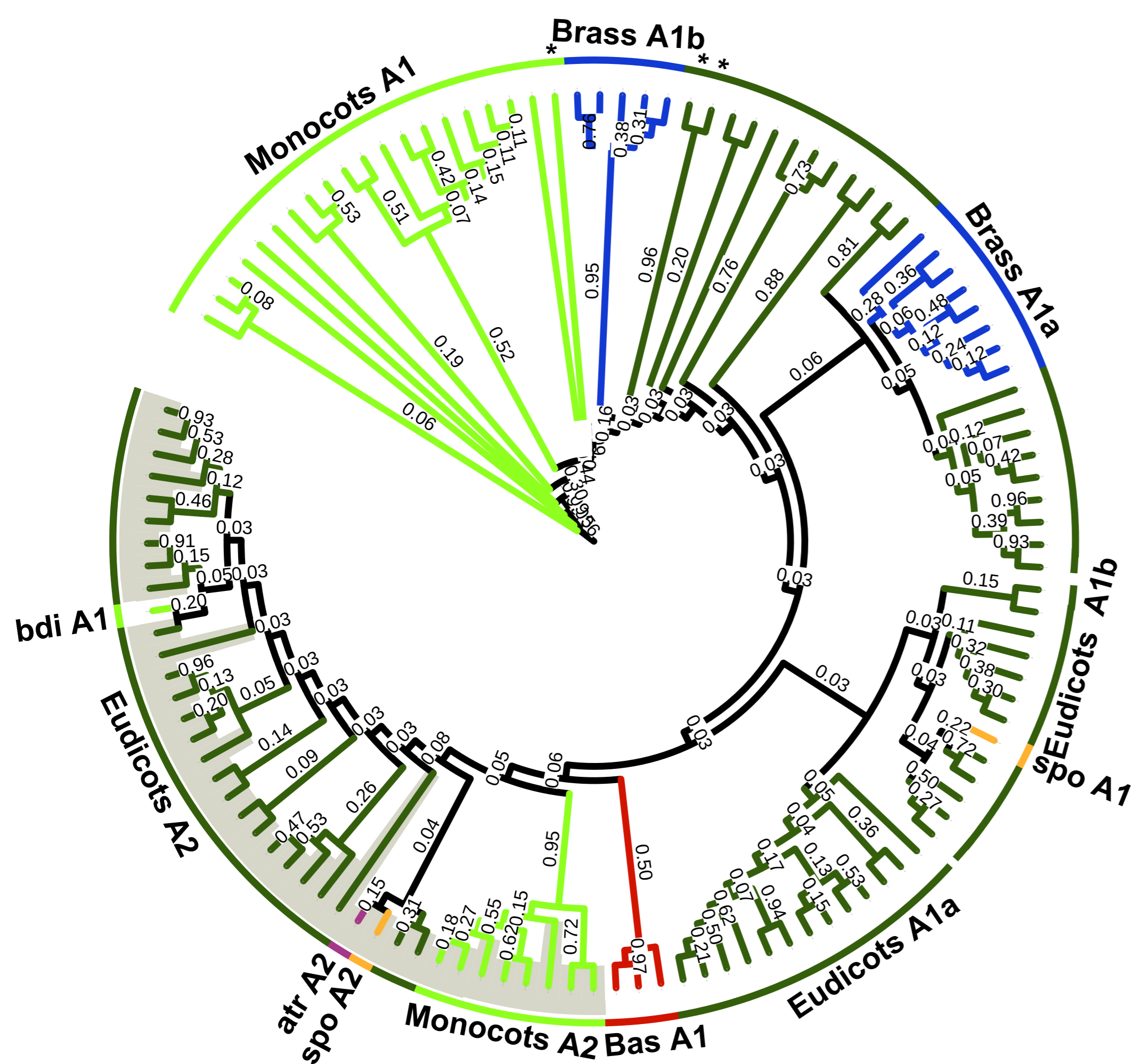

PAE1 LOGO MP

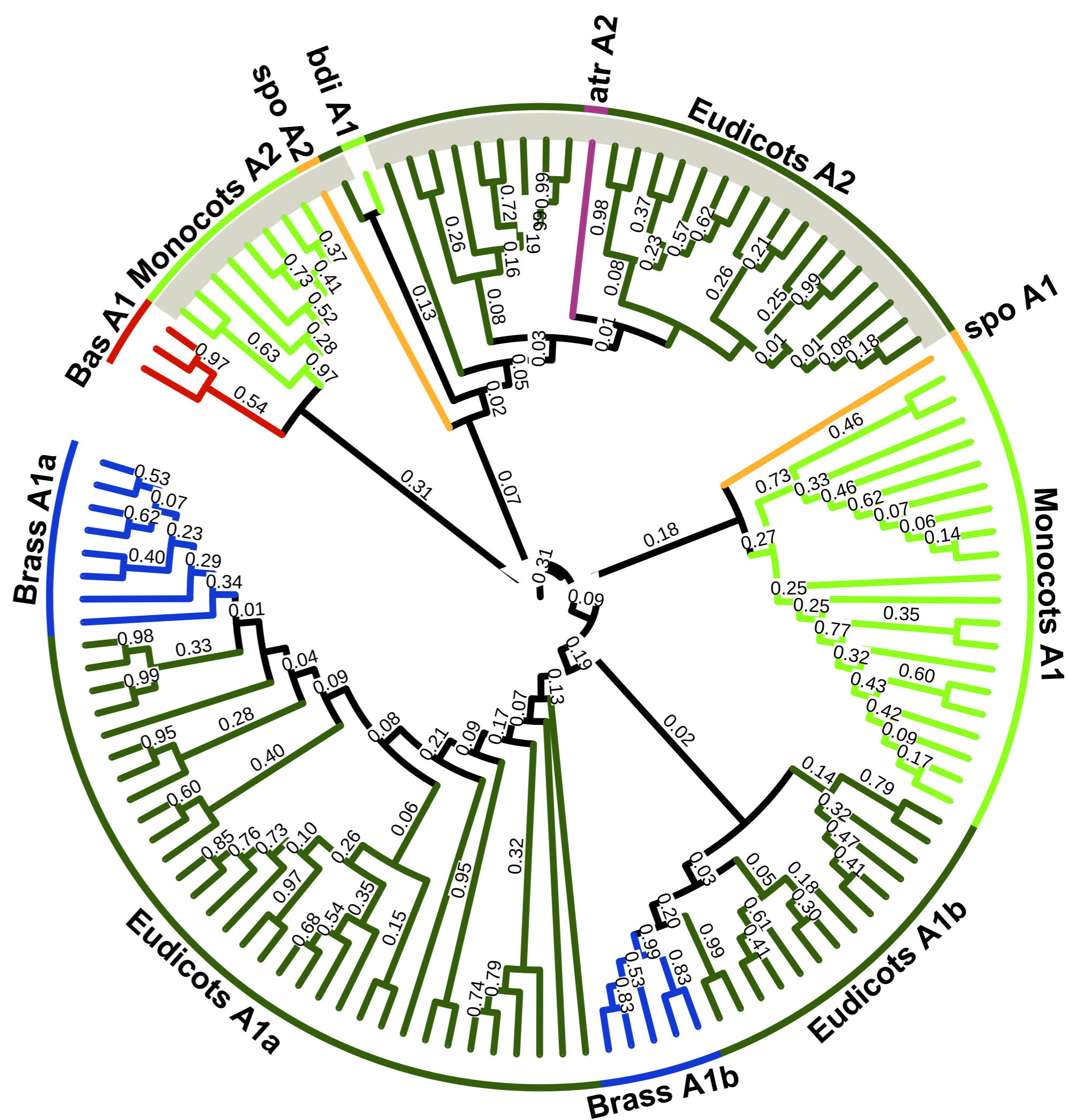

PAE1 LOGO ML

Supplement: Additional file 6: — Circular phylogenetic tree of class A CIDs proteins based on the PAE1 LOGO (LOGO #A1), or on the complete polypeptide sequence. The topology was generated using the maximum-parsimony (MP) or the maximum-likelihood (ML), as indicated (see Methods). (*) are in dissimilar places in both MP trees. Color codes of branches are as depicted in Fig. 4. Labels on branches are based on the NJ trees from Fig. 4. (PDF 18177 kb) [file 12862_2015_475_MOESM6_ESM.pdf]

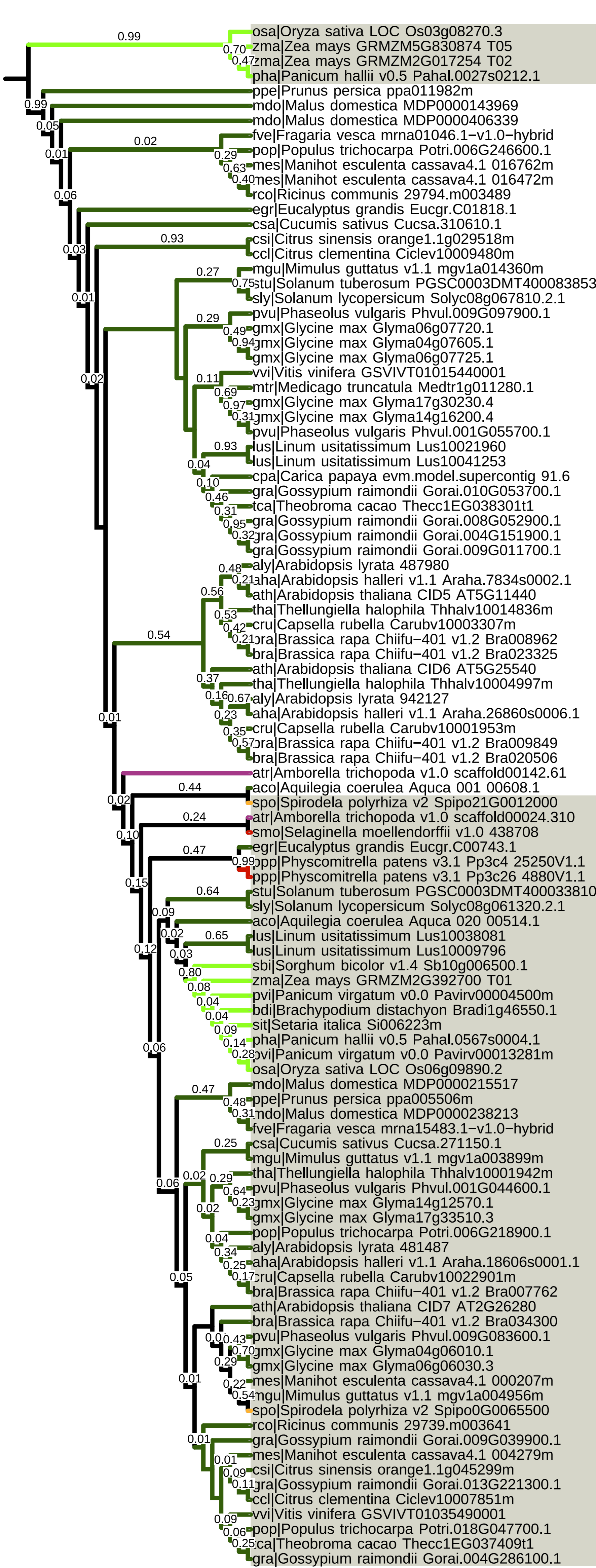

complete sequence

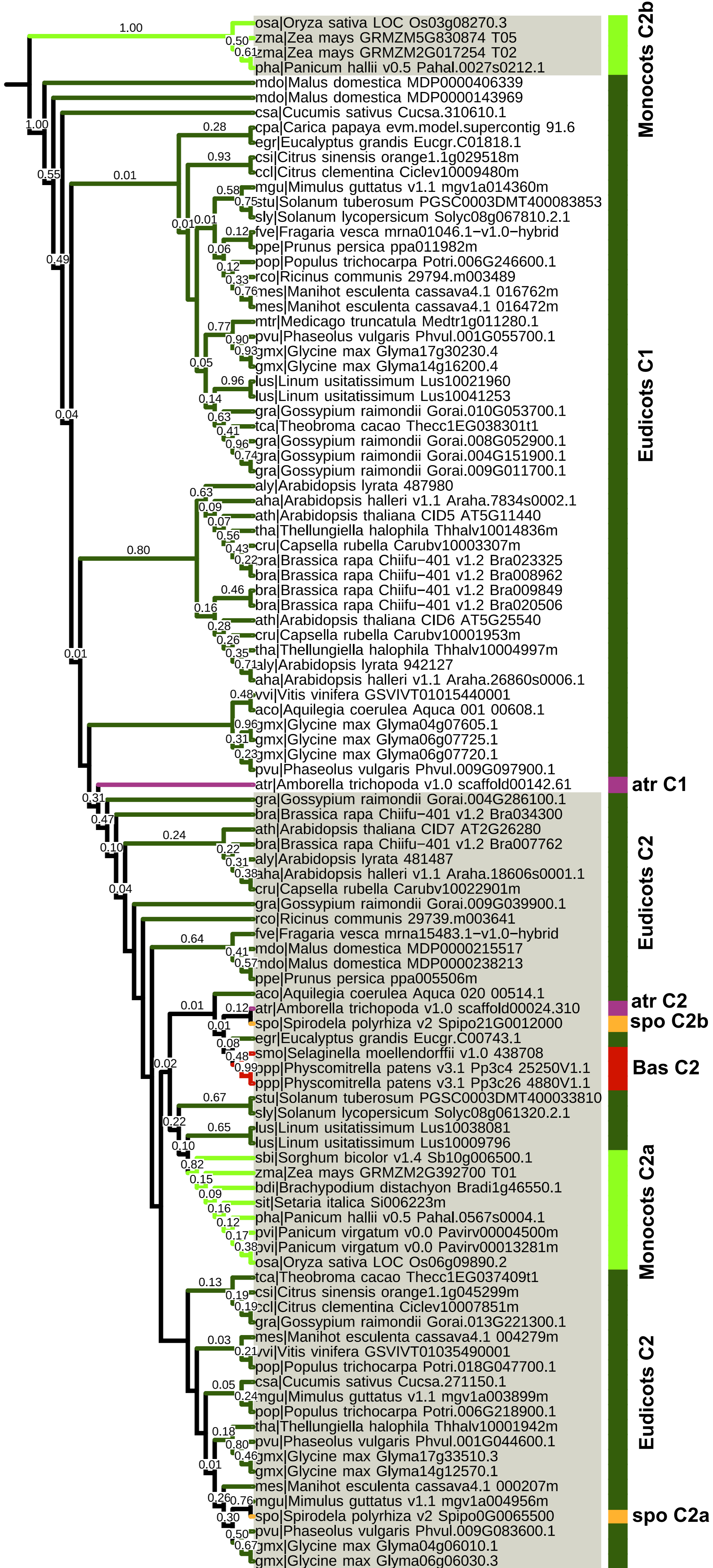

CUE LOGO

Supplement: Additional file 9: — Rectangular phylogenetic tree of class C CIDs proteins based on the CUE motif (LOGO #C1), or on the complete polypeptide sequence. The topology was generated using the neighbor-joining (NJ) method. Species and gene names are those presented in Additional file 1. The color codes on the branches for groups of organisms are the same as in Fig. 4; protein names are displayed. (PDF 16124 kb) [file 12862_2015_475_MOESM9_ESM.pdf]

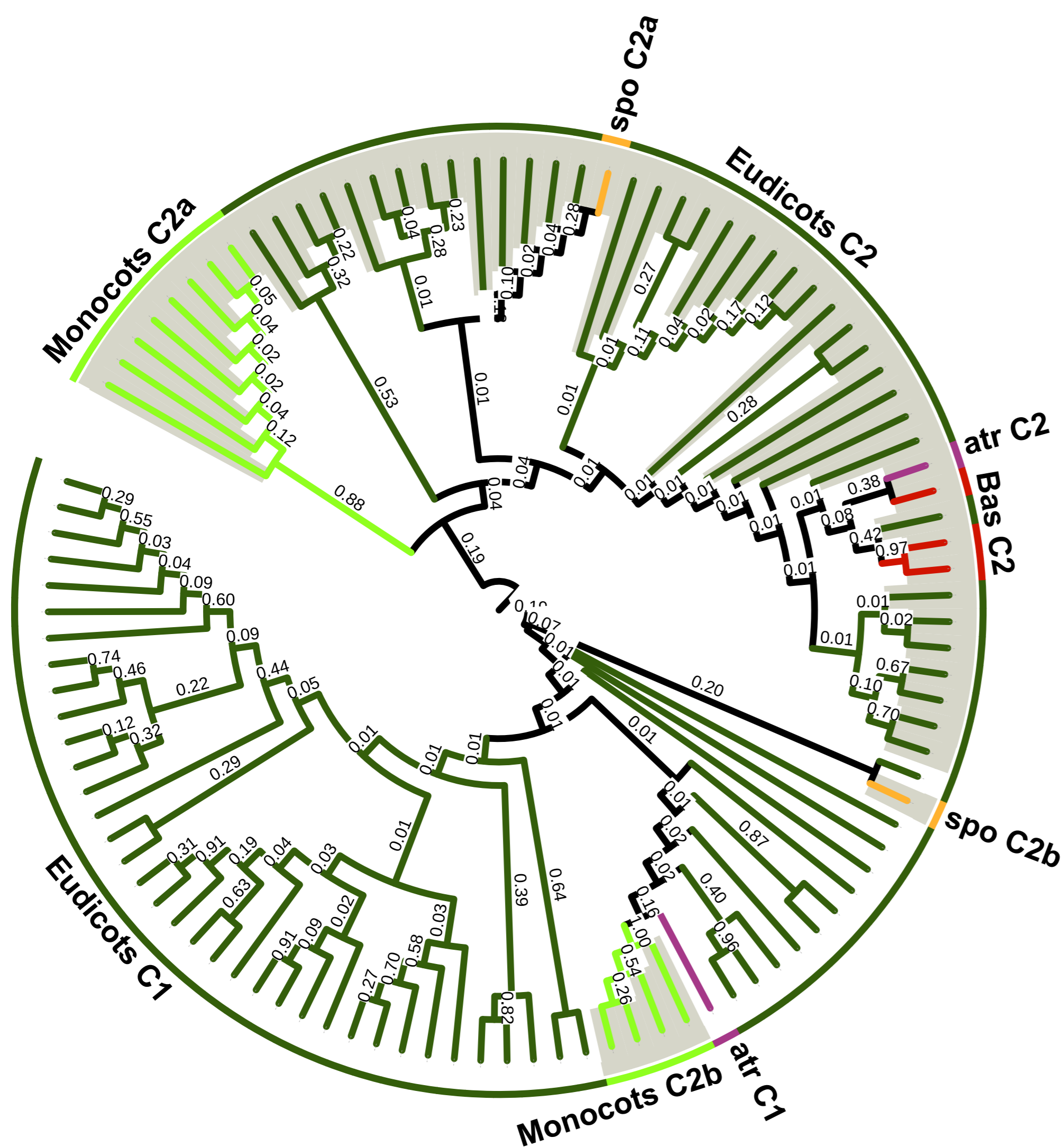

complete sequence MP

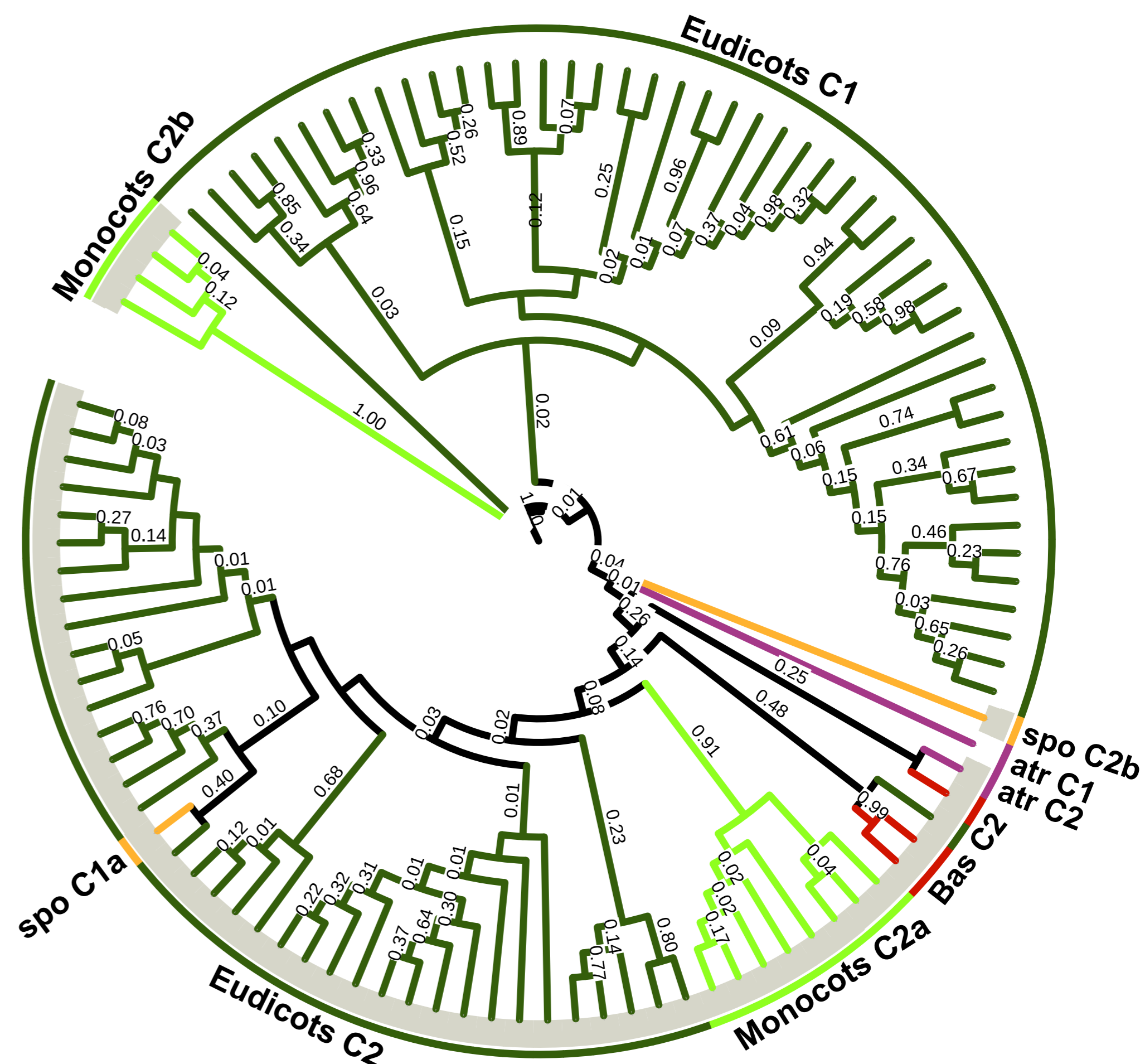

complete sequence ML

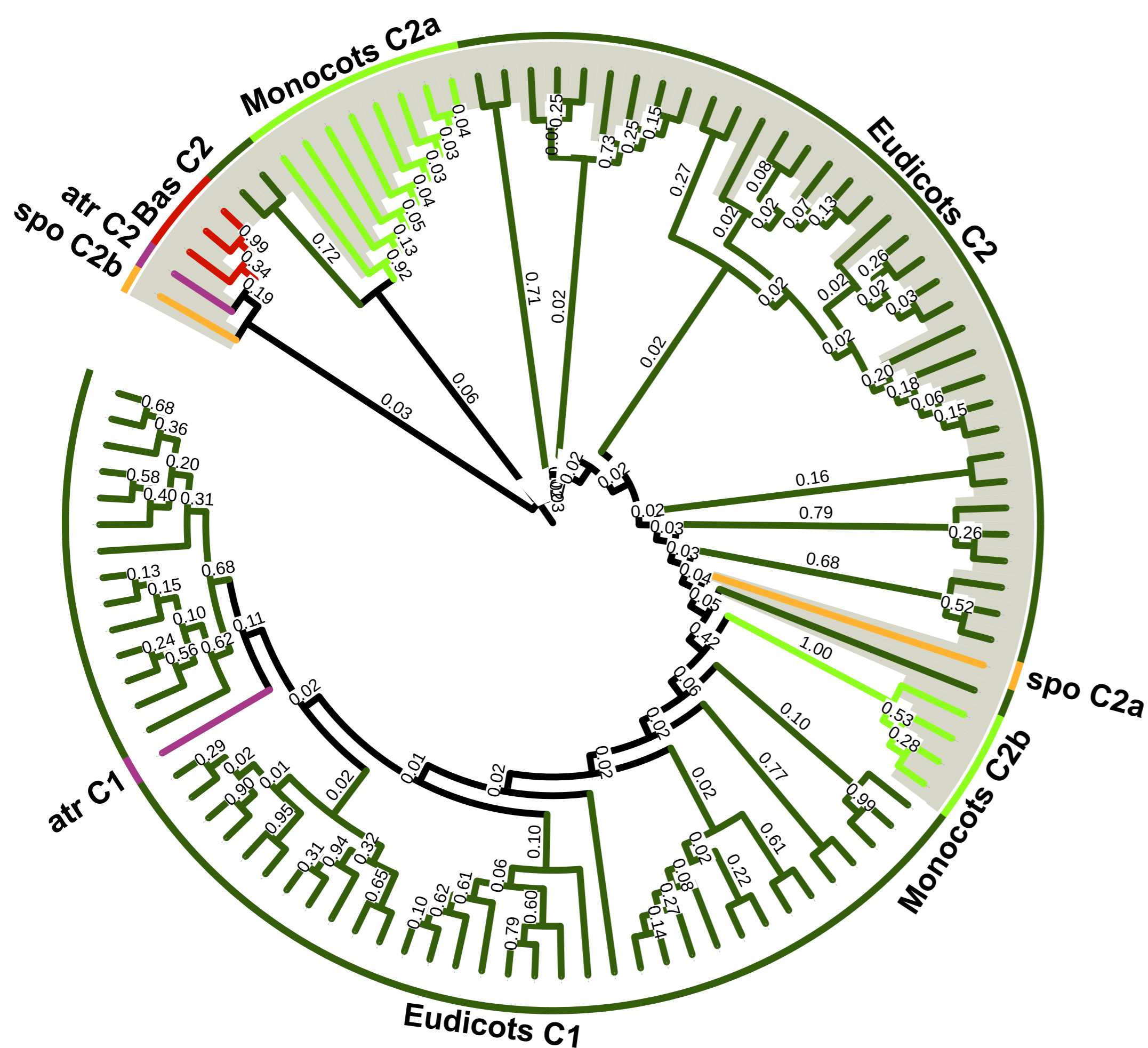

CUE LOGO MP

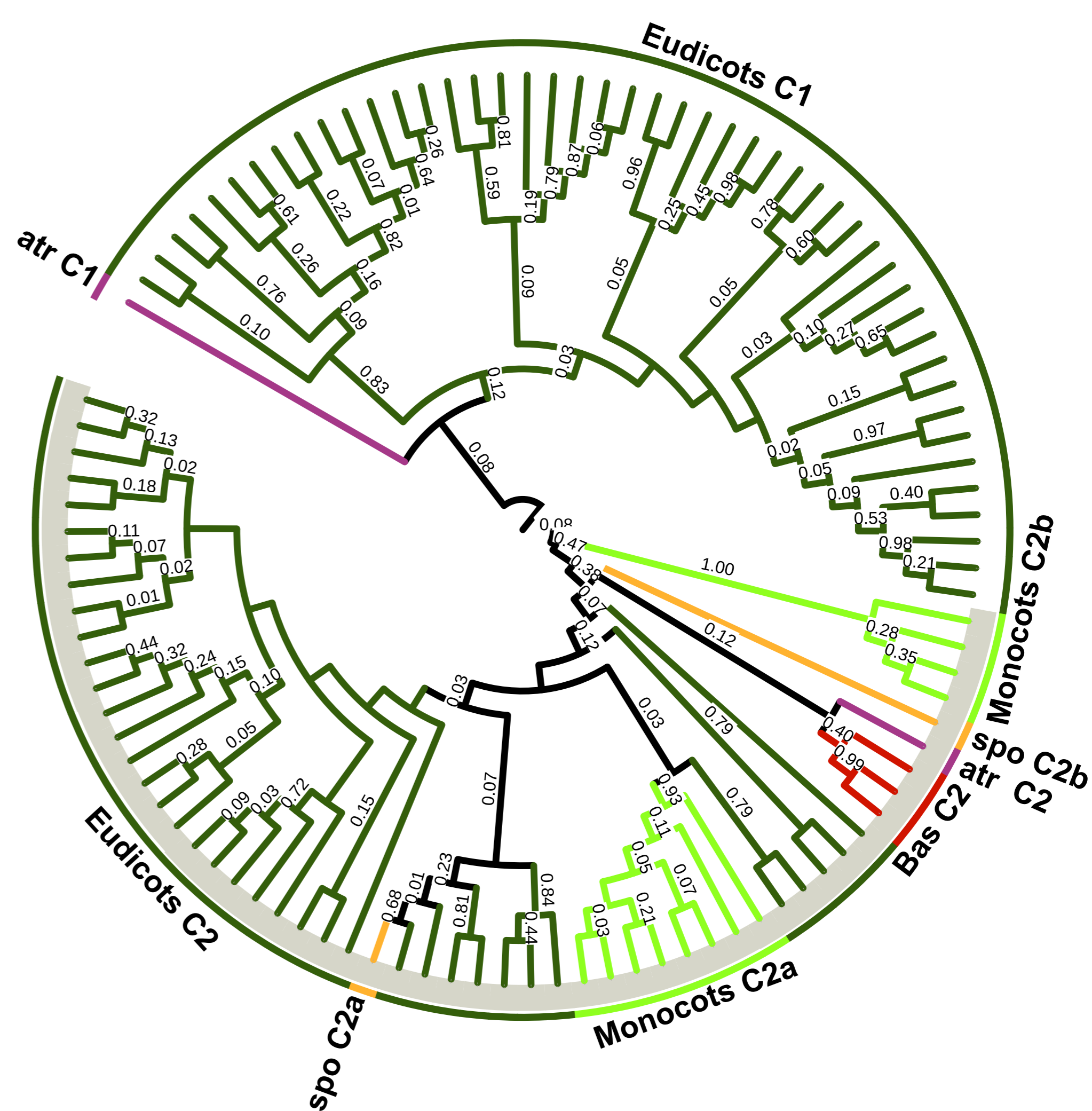

CUE LOGO MP

Supplement: Additional file 10: — Circular phylogenetic tree of class C CIDs proteins based on the CUE motif (LOGO #C1), or on the complete polypeptide sequence. The topology was generated using the MP or the ML methods, as indicated (see Methods). Color codes of branches are as depicted in Fig. 4. Labels of branches are based on the NJ trees from Fig. 7. (PDF 9581 kb) [file 12862_2015_475_MOESM10_ESM.pdf]

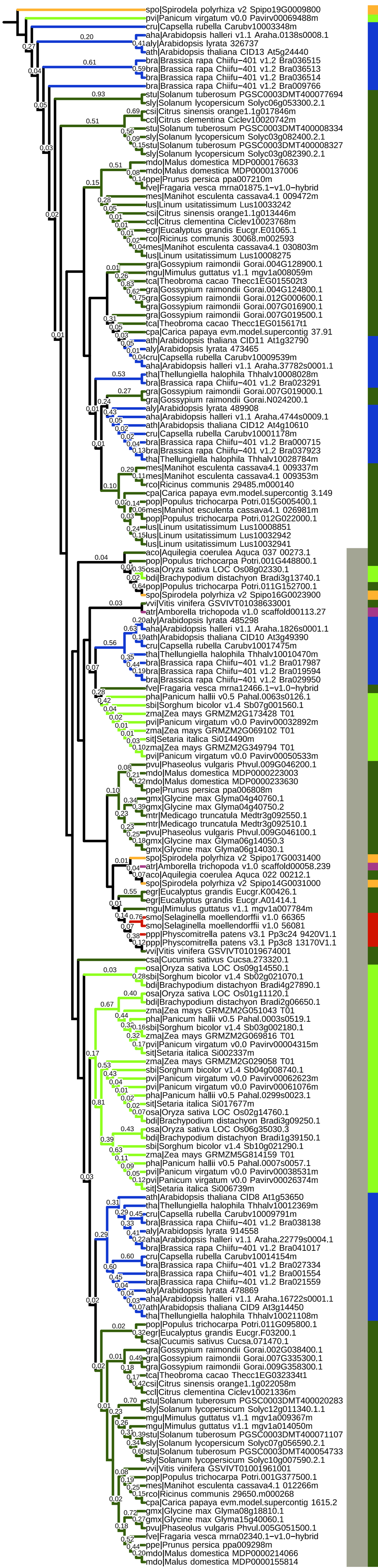

complete sequence

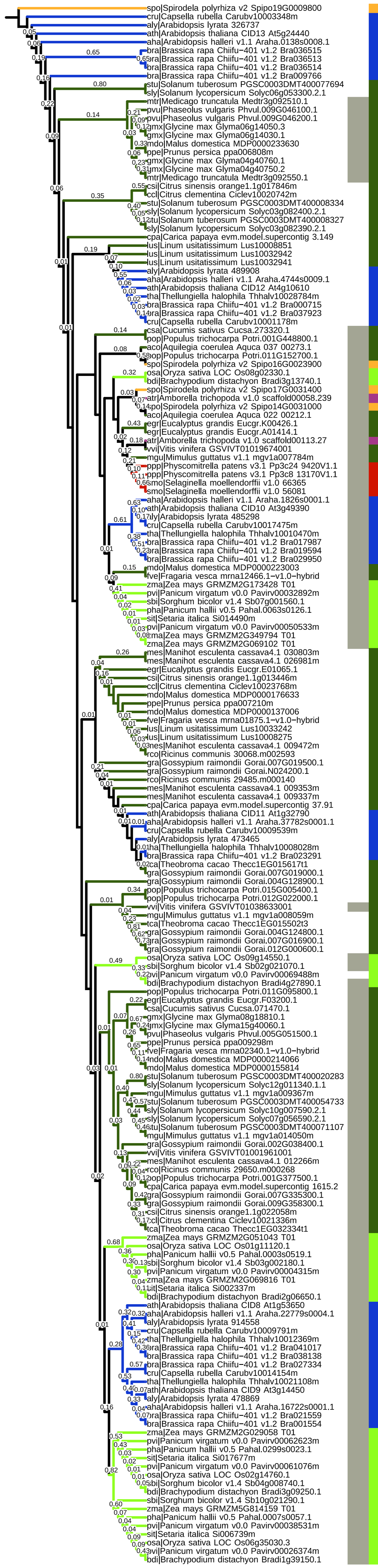

RRM1-RRM2 LOGO

Supplement: Additional file 11: — Rectangular phylogenetic tree of class D CIDs proteins based on RRM1 and RRM2 motifs, or on the complete polypeptide sequence, as described in Fig. 9 . (PDF 27070 kb) [file 12862_2015_475_MOESM11_ESM.pdf]
