# Supplementary material for: Evolutionary history exposes radical diversification among classes of interaction partners of the MLLE domain of plant poly(A)-binding proteins
Source: BMC Evol Biol. 2015 Sep 16;15:195. doi: 10.1186/s12862-015-0475-1 (PMC4574140; doi:10.1186/s12862-015-0475-1)
Supplement: Additional file 7: — Protein sequence alignment for class C sequence LOGO #C8. The sequence alignments were performed using ClustalX 2.0.12; default colors were used. (PDF 14808 kb) [file 12862_2015_475_MOESM7_ESM.pdf]

class C1

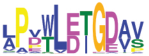

bra	Brassica_rapa_Chiifu-401_v1.2_Bra007762	PVAWLETGDAV
gra	Gossypium_raimondii_Gorai.013G221300.1	--AWLETGDAV
aly	Arabidopsis_lyrata_481487	APVWVETGDAV
aha	Arabidopsis_halleri_v1.1_Araha.18606s0001.1	APVWVETGDAV
ath	Arabidopsis_thaliana_CID7_AT2G26280	APVWVETGDAV
cru	Capsella_rubella_Carubv10022901m	APVWVETGDAV
tha	Thellungiella_halophila_Thhalv10001942m	APVWVETGDAV
gmx	Glycine_max_Glyma04g06010.1	APVWLETSDAV
tca	Theobroma_cacao_Thecc1EG037409t1	APVWLETGDAV
gra	Gossypium_raimondii_Gorai.009G039900.1	APVWLETGDAV
fve	Fragaria_vesca_mrna15483.1-v1.0-hybrid	APVWLETGDAV
ppe	Prunus_persica_ppa005506m	APVWLETGDAV
mdo	Malus_domestica_MDP0000215517	APVWLETGDAV
mdo	Malus_domestica_MDP0000238213	APVWLETGDAV
pvu	Phaseolus_vulgaris_Phvul.001G044600.1	APVWLETGDAV
gmx	Glycine_max_Glyma17g33510.3	APVWLETGDAV
gmx	Glycine_max_Glyma14g12570.1	APVWLETGDAV
bra	Brassica_rapa_Chiifu-401_v1.2_Bra034300	APVWLETGDAV
csa	Cucumis_sativus_Cucsa.271150.1	APVWLETGEAV
mes	Manihot_esculenta_cassava4.1_004279m	APVWLETGEAV
rco	Ricinus_communis_29739.m003641	APVWLETGEAV
pop	Populus_trichocarpa_Potri.018G047700.1	APVWLETGEAV
pop	Populus_trichocarpa_Potri.006G218900.1	APVWLETGEAV
csi	Citrus_sinensis_orange1.1g045299m	APVWLETGEAV
ccl	Citrus_clementina_Ciclev10007851m	APVWLETGEAV
aco	Aquilegia_coerulea_Aquca_020_00514.1	APVWLETGEAV
stu	Solanum_tubberosum_PGSC0003DMP400023014	APVWLETGEAV
sly	Solanum_lycopersicum_Solyc08g061320.2.1	APVWLETGEAV
lus	Linum_usitatissimum_Lus10038081	APTWLETGEAV
lus	Linum_usitatissimum_Lus10009796	APTWLETGEAV
vvi	Vitis_vinifera_GSVIVT01035490001	APAWVETGEAV
mgu	Mimulus_guttatus_v1.1_mgv1a003899m	SPIWLETGEAV
mgu	Mimulus_guttatus_v1.1_mgv1a004956m	SPVWLETGEAV
smo	Selaginella_moellendorffii_438708	QP-WLETGEAV
ppp	Physcomitrella_patens_Pp1s159_78V6.1	VP-WLETGESV
egr	Eucalyptus_grandis_Eucgr.C00743.1	TPVWLETGEAV
pvu	Phaseolus_vulgaris_Phvul.009G083600.1	APIWLETGDAV
gmx	Glycine_max_Glyma06g06030.3	FPVWLETGDAV
sbi	Sorgum_bicolor_Sb10g006500.1	AP-WLETGDAV
zma	Zea_mays_GRMZM2G392700_T01	AP-WLETGDAV
pvi	Panicum_virgatum_v0.0_Pavirv00004500m	AP-WLETGDAV
pvi	Panicum_virgatum_v0.0_Pavirv00013281m	AP-WLETGDAV
pha	Panicum_hallii_v0.5_Pahal.0567s0004.1	AP-WLETGDAV
sit	Setaria_italica_Si006223m	AP-WLETGDAV
osa	Oriza_sativa_LOC_Os06g09890.2	AP-WLETGDAV
atr	Amborella_trichopoda_scaffold00024.310	AP-WLETGDTV
bdi	Brachypodium_distachyon_Bradi1g46550.1	AP-WLDTGDAV
gra	Gossypium_raimondii_Gorai.004G286100.1	A-----TGDAV
mes	Manihot_esculenta_cassava4.1_000207m	VPAWLETGAAV
zma	Zea_mays_GRMZM5G830874_T05	GPAWLESDAE-
pha	Panicum_hallii_v0.5_Pahal.0027s0212.1	GPAWLENDAA-
osa	Oriza_sativa_LOC_Os03g08270.3	GPAWLEADTA-
zma	Zea_mays_GRMZM2G017254_T02	GAAWLETDAA-

class C2

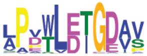

aly	Arabidopsis_lyrata_487980	LPETLDIGDIS
aha	Arabidopsis_halleri_v1.1_Araha.7834s0002.1	LPETLDIGDIS
ath	Arabidopsis_thaliana_CID5_AT5G25540	LPETLDIGDIS
cru	Capsella_rubella_Carubv10003307m	LPETLDIGDIS
tha	Thellungiella_halophila_Thhalv10014836m	LPETLDIEDIS
bra	Brassica_rapa_Chiifu-401_v1.2_Bra008962	LPETLDIGVMC
bra	Brassica_rapa_Chiifu-401_v1.2_Bra023325	LPETLDIGDLC
lus	Linum_usitatissimum_Lus10021960	LPDTLEIGDVP
lus	Linum_usitatissimum_Lus10041253	LPDTLEIGDVP
aly	Arabidopsis_lyrata_910614	LPDTLDIGDVP
aha	Arabidopsis_halleri_v1.1_Araha.26860s0006.1	LPDTLDIGDVP
ath	Arabidopsis_thaliana_CID6_AT5G11440	LPDTLDIGDVP
cru	Capsella_rubella_Carubv10001953m	LPDTLDIGDVP
bra	Brassica_rapa_Chiifu-401_v1.2_Bra009849	LPDTLDIGDVP
tha	Thellungiella_halophila_Thhalv10004997m	LPDTLDIGDVP
bra	Brassica_rapa_Chiifu-401_v1.2_Bra020506	LPDTLDIGDVP
aco	Aquilegia_coerulea_Aquca_001_00608.1	LPDTLDIGDVP
cpa	Carica_papaya_evm.model.supercontig_91.6	LPDTLDIGDVP
pop	Populus_trichocarpa_Potri.006G246600.1	LPDTLDIGDVY
tca	Theobroma_cacao_Thecc1EG038301t1	LPDTLDIGDVS
ccl	Citrus_clementina_Ciclev10009480m	LPDTLDIGDVS
csi	Citrus_sinensis_orange1.1g029518m	LPDTLDIGDVS
fve	Fragaria_vesca_mrna01046.1-v1.0-hybrid	LPDTLDIGDVS
ppe	Prunus_persica_ppa011982m	LPDTLDIGDVS
mdo	Malus_domestica_MDP0000143969	LPDTLDIGDVS
mdo	Malus_domestica_MDP0000406339	LPDTLDIGDVS
rco	Ricinus_communis_29794.m003489	LPDTLDIGDVS
mes	Manihot_esculenta_cassava4.1_016762m	LPDTLDIGDVS
mgu	Mimulus_guttatus_v1.1_mgv1a014360m	LPDTLDIGDVS
mes	Manihot_esculenta_cassava4.1_016472m	LPDTLDIGDIS
gra	Gossypium_raimondii_Gorai.008G052900.1	LPDTLDIGDIS
gra	Gossypium_raimondii_Gorai.004G151900.1	LPDTLDIGDIS
csa	Cucumis_sativus_Cucsa.310610.1	LPDTLDIGDIS
gmx	Glycine_max_Glyma06g07720.1	--KTLDIGSVS
stu	Solanum_tuberosum_PGSC0003DMP400056115	LPDSLDIGDVS
sly	Solanum_lycopersicum_Solyc08g067810.2.1	LPDSLDIGDVS
vvi	Vitis_vinifera_GSVIVT01015440001	LPESLDIGDVS
gmx	Glycine_max_Glyma17g30230.4	LPETLDIGDVS
gmx	Glycine_max_Glyma14g16200.4	LPETLDIGDVS
pvu	Phaseolus_vulgaris_Phvul.001G055700.1	LPETLDIGDVS
egr	Eucalyptus_grandis_Eucgr.C01818.1	LPETLDIGDVA
atr	Amborella_trichopoda_scaffold00142.61	LPTELAISGTS
