# Supplementary material for: Evolutionary history exposes radical diversification among classes of interaction partners of the MLLE domain of plant poly(A)-binding proteins
Source: BMC Evol Biol. 2015 Sep 16;15:195. doi: 10.1186/s12862-015-0475-1 (PMC4574140; doi:10.1186/s12862-015-0475-1)
Supplement: Additional file 8: — Alignment of the CUE motif of CID C proteins. The two CID C classes and the Brassicaceae species are indicated by brackets. The region encompassing sequence LOGO #1 from all CID C sequences was aligned using ClustalX 2.0.12 software, and a default color code was applied. The locations of important residues are highlighted by arrows (the invariant proline residue and a di-leucine motif). (*) indicates substitution of the invariant proline residue, (**) indicates substitution at the di-leucine motif. (PDF 4954 kb) [file 12862_2015_475_MOESM8_ESM.pdf]

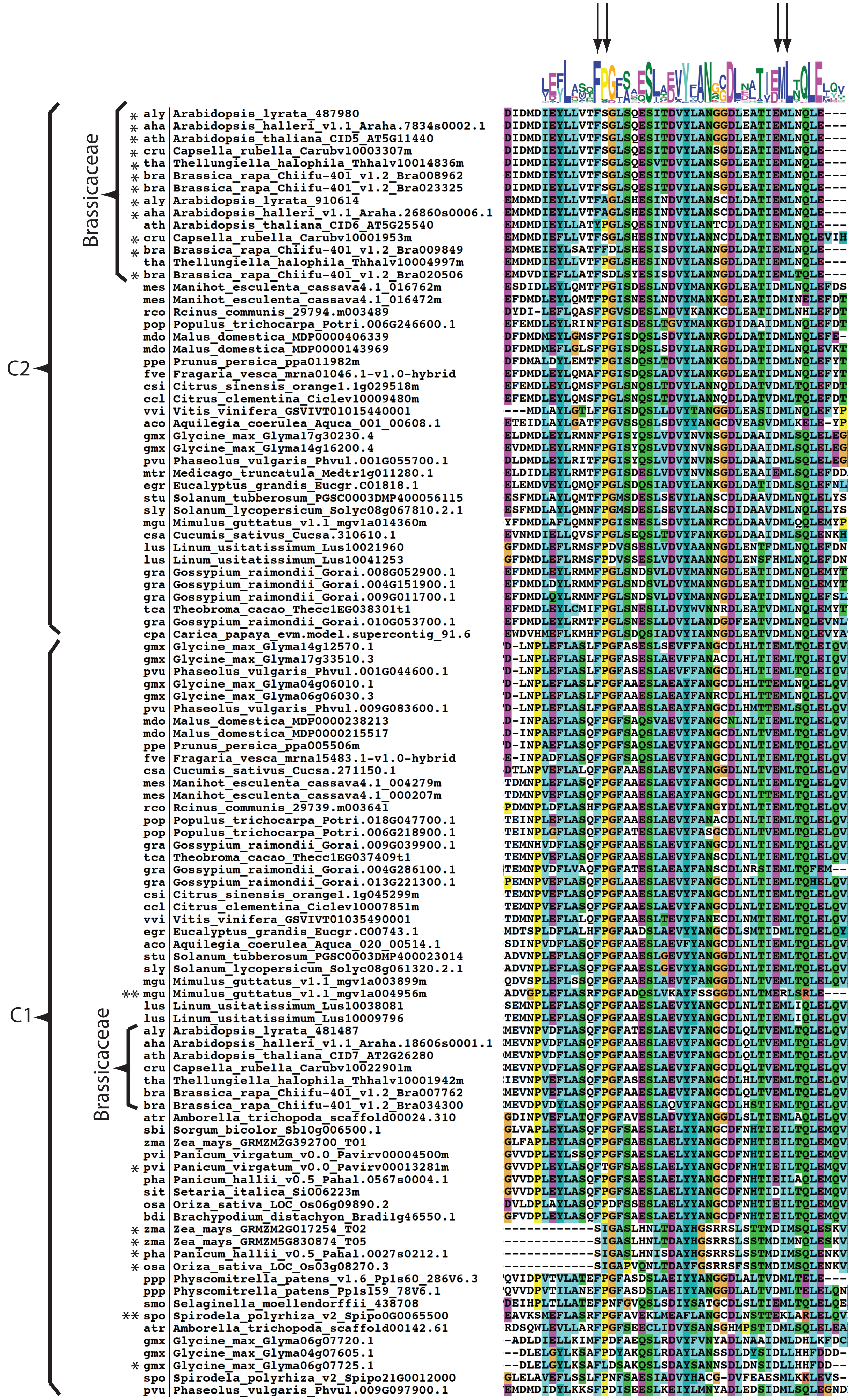

C2
C1
Brassicaceae
Brassicaceae
* aly Arabidopsis_lyrata_487980
* aha Arabidopsis_halleri_v1.1_Araha.7834s0002.1
* ath Arabidopsis_thaliana_CID5_AT5G11440
* cru Capsella_rubella_Carubv10003307m
* tha Thellungiella_halophila_Thhalv10014836m
* bra Brassica_rapa_Chiifu-401_v1.2_Bra008962
* bra Brassica_rapa_Chiifu-401_v1.2_Bra023325
* aly Arabidopsis_lyrata_910614
* aha Arabidopsis_halleri_v1.1_Araha.26860s0006.1
ath Arabidopsis_thaliana_CID6_AT5G25540
* cru Capsella_rubella_Carubv10001953m
* bra Brassica_rapa_Chiifu-401_v1.2_Bra009849
tha Thellungiella_halophila_Thhalv10004997m
* bra Brassica_rapa_Chiifu-401_v1.2_Bra020506
mes Manihot_esculenta_cassava4.1_016762m
mes Manihot_esculenta_cassava4.1_016472m
rco Rcinus_communis_29794.m003489
pop Populus_trichocarpa_Potri.006G246600.1
mdo Malus_domestica_MDP0000406339
mdo Malus_domestica_MDP0000143969
ppe Prunus_persica_ppa011982m
fve Fragaria_vesca_mrna01046.1-v1.0-hybrid
csi Citrus_sinensis_orange1.1g029518m
ccl Citrus_clementina_Ciclev10009480m
vvi Vitis_vinifera_GSVIVT01015440001
aco Aquilegia_coerulea_Aquca_001_00608.1
gmx Glycine_max_Glyma17g30230.4
gmx Glycine_max_Glyma14g16200.4
pvu Phaseolus_vulgaris_Phvul.001G055700.1
mtr Medicago_truncatula_Medtr1g011280.1
egr Eucalyptus_grandis_Eucgr.C01818.1
stu Solanum_tubberosum_PGSC0003DMP400056115
sly Solanum_lycopersicum_Solyc08g067810.2.1
mgu Mimulus_guttatus_v1.1_mgv1a014360m
csa Cucumis_sativus_Cucsa.310610.1
lus Linum_usitatissimum_Lus10021960
lus Linum_usitatissimum_Lus10041253
gra Gossypium_raimondii_Gorai.008G052900.1
gra Gossypium_raimondii_Gorai.004G151900.1
gra Gossypium_raimondii_Gorai.009G011700.1
tca Theobroma_cacao_Thecc1EG038301t1
gra Gossypium_raimondii_Gorai.010G053700.1
cpa Carica_papaya_evm.model.supercontig_91.6
gmx Glycine_max_Glyma14g12570.1
gmx Glycine_max_Glyma17g33510.3
pvu Phaseolus_vulgaris_Phvul.001G044600.1
gmx Glycine_max_Glyma04g06010.1
gmx Glycine_max_Glyma06g06030.3
pvu Phaseolus_vulgaris_Phvul.009G083600.1
mdo Malus_domestica_MDP0000238213
mdo Malus_domestica_MDP0000215517
ppe Prunus_persica_ppa005506m
fve Fragaria_vesca_mrna15483.1-v1.0-hybrid
csa Cucumis_sativus_Cucsa.271150.1
mes Manihot_esculenta_cassava4.1_004279m
mes Manihot_esculenta_cassava4.1_000207m
rco Rcinus_communis_29739.m003641
pop Populus_trichocarpa_Potri.018G047700.1
pop Populus_trichocarpa_Potri.006G218900.1
gra Gossypium_raimondii_Gorai.009G039900.1
tca Theobroma_cacao_Thecc1EG037409t1
gra Gossypium_raimondii_Gorai.004G286100.1
gra Gossypium_raimondii_Gorai.013G221300.1
csi Citrus_sinensis_orange1.1g045299m
ccl Citrus_clementina_Ciclev10007851m
vvi Vitis_vinifera_GSVIVT01035490001
egr Eucalyptus_grandis_Eucgr.C00743.1
aco Aquilegia_coerulea_Aquca_020_00514.1
stu Solanum_tubberosum_PGSC0003DMP400023014
sly Solanum_lycopersicum_Solyc08g061320.2.1
mgu Mimulus_guttatus_v1.1_mgv1a003899m
** mgu Mimulus_guttatus_v1.1_mgv1a004956m
lus Linum_usitatissimum_Lus10038081
lus Linum_usitatissimum_Lus10009796
aly Arabidopsis_lyrata_481487
aha Arabidopsis_halleri_v1.1_Araha.18606s0001.1
ath Arabidopsis_thaliana_CID7_AT2G26280
cru Capsella_rubella_Carubv10022901m
tha Thellungiella_halophila_Thhalv10001942m
bra Brassica_rapa_Chiifu-401_v1.2_Bra007762
bra Brassica_rapa_Chiifu-401_v1.2_Bra034300
atr Amborella_trichopoda_scaffold00024.310
sbi Sorgum_bicolor_Sb10g006500.1
zma Zea_mays_GRMZM2G392700_T01
pvi Panicum_virgatum_v0.0_Pavirv00004500m
* pvi Panicum_virgatum_v0.0_Pavirv00013281m
pha Panicum_hallii_v0.5_Pahal.0567s0004.1
sit Setaria_italica_Si006223m
osa Oriza_sativa_LOC_Os06g09890.2
bdi Brachypodium_distachyon_Bradi1g46550.1
* zma Zea_mays_GRMZM2G017254_T02
* zma Zea_mays_GRMZM5G830874_T05
* pha Panicum_hallii_v0.5_Pahal.0027s0212.1
* osa Oriza_sativa_LOC_Os03g08270.3
ppp Physcomitrella_patens_v1.6_Pp1s60_286V6.3
ppp Physcomitrella_patens_Pp1s159_78V6.1
smo Selaginella_moellendorffii_438708
** spo Spirodela_polyrhiza_v2_Spipo0G0065500
atr Amborella_trichopoda_scaffold00142.61
gmx Glycine_max_Glyma06g07720.1
gmx Glycine_max_Glyma04g07605.1
* gmx Glycine_max_Glyma06g07725.1
spo Spirodela_polyrhiza_v2_Spipo21G0012000
pvu Phaseolus_vulgaris_Phvul.009G097900.1
